# Modeling and Re-Engineering of *Azotobacter vinelandii* Alginate Lyase to Enhance Its Catalytic Efficiency for Accelerating Biofilm Degradation

**DOI:** 10.1371/journal.pone.0156197

**Published:** 2016-06-02

**Authors:** Chul Ho Jang, Yu Lan Piao, Xiaoqin Huang, Eun Jeong Yoon, So Hee Park, Kyoung Lee, Chang-Guo Zhan, Hoon Cho

**Affiliations:** 1 Department of Otolaryngology, Chonnam National University Medical School, Gwangju, 501–757, Republic of Korea; 2 Department of Polymer Science & Engineering, Chosun University, Gwangju, 501–759, South Korea; 3 Molecular Modeling and Biopharmaceutical Center and Department of Pharmaceutical Sciences, College of Pharmacy, University of Kentucky, 789 South Limestone Street, Lexington, Kentucky, 40536, United States of America; 4 Department of Microbiology, Changwon National University, Changwon, Kyongnam, 641–773, Republic of Korea; Universidad de Santiago de Compostela, SPAIN

## Abstract

Alginate is known to prevent elimination of *Pseudomonas aeruginosa* biofilms. Alginate lyase (AlgL) might therefore facilitate treatment of *Pseudomonas aeruginosa*-infected cystic fibrosis patients. However, the catalytic activity of wild-type AlgL is not sufficiently high. Therefore, molecular modeling and site-directed mutagenesis of AlgL might assist in enzyme engineering for therapeutic development. AlgL, isolated from *Azotobacter vinelandii*, catalyzes depolymerization of alginate via a β-elimination reaction. AlgL was modeled based on the crystal structure template of *Sphingomonas* AlgL species A1-III. Based on this computational analysis, AlgL was subjected to site-directed mutagenesis to improve its catalytic activity. The *k*_cat_/*K*_m_ of the K194E mutant showed a nearly 5-fold increase against the acetylated alginate substrate, as compared to the wild-type. Double and triple mutants (K194E/K245D, K245D/K319A, K194E/K245D/E312D, and K194E/K245D/K319A) were also prepared. The most potent mutant was observed to be K194E/K245D/K319A, which has a 10-fold improved k_cat_ value (against acetylated alginate) compared to the wild-type enzyme. The antibiofilm effect of both AlgL forms was identified in combination with piperacillin/tazobactam (PT) and the disruption effect was significantly higher in mutant AlgL combined with PT than wild-type AlgL. However, for both the wild-type and K194E/K245D/K319A mutant, the use of the AlgL enzyme alone did not show significant antibiofilm effect.

## Introduction

Biofilms formed by adherent microorganisms are composed of a self-produced polymer matrix, consisting of proteins, polysaccharides, and nucleic acids [1]. Polysaccharides play a crucial role in the formation of *Pseudomonas aeruginosa* biofilms. A number of previous studies have indicated that *Pseudomonas aeruginosa* mutants deficient in the ability to synthesize exopolysaccharides (EPS) are unable to form biofilms [2]. The bacterium *P*. *aeruginosa* is an opportunistic pathogen par excellence, which causes severe and life-threatening infections in various organs of immune-compromised hosts, including the oral cavity [3], heart valves (endocarditis) [4], lungs of patients with cystic fibrosis (CF; causing chronic bronchopneumonia) [5], and the middle ear of patients with persistent otitis media [6]. Patients with chronic rhinosinusitis [7], chronic osteomyelitis and prosthetic joint infections [8–10], and chronic wounds [11, 12] are also affected. In these cases, the bacterium is transmitted via intravenous catheters and stents [13]. Such infections are extremely difficult to control as *P*. *aeruginosa* adopts a biofilm mode of growth, rendering the bacteria resistant to antibiotics and phagocytic cells. During the course of infection, *P*. *aeruginosa* usually undergoes a phenotypic conversion to a mucoid colony, characterized by the overproduction of the exopolysaccharide, alginate. Nowadays, the prevalence antibiotic resistant *P*. *aeruginosa* such as *ciprofloxacin-resistant P*. *aeruginosa* (CRPA) is increasing, especially in cases of chronic otitis media or keratitis due to use of topical ciprofloxacin drops [14].

Generally, the polysaccharide lyases (PL) can be classified into 14 families (PL-1 to PL-14) based on their primary structures [15]. The *Azotobacter vinelandii* alginate lyase (AvAlgL) and the *Pseudomonas aeruginosa* alginate lyase (PaAlgL) belong to the subfamily 7 (PL-7). Alginate functions as an extracellular matrix material, enabling the formation of differentiated biofilms. These reduce the diffusion of clinical antibiotics and protect the embedded cells against human antibacterial defense mechanisms [16, 17]. Nivens et al. [16] suggested that alginate contributes to biofilm architecture. Hay et al. [18] reported that the finger-like micro-colonies present in mucoid strain biofilms were completely absent in the alginate-negative mutant biofilms, indicating that alginate is essential for the formation of these biofilm structures. Other studies have shown that further increase in the levels of alginate production leads to formation of extremely large micro-colonies. This demonstrates the important role of this exopolysaccharide in micro-colony formation.

Alginate can be degraded by alginate lyases (AlgLs), which catalyze β-elimination of the 4-O-linked glycosidic bond, resulting in formation of unsaturated uronic acid-containing oligosaccharides. Therefore, AlgLs catalyze the removal of EPS from the surface of mucoid strains of *Pseudomonas aeruginosa* [19] and reduce their attachment ability [20]. Alkawash et al. [21] reported that co-administration of AlgL with antibiotics significantly increased the killing of *in vitro* mucoid *P*. *aeruginosa* biofilms, grown in conditions similar to those found in the CF respiratory tract. Bayer et al. [19] also discovered that co-administration of AlgL with amikacin caused negation of a larger amount of vegetative culture, compared to administration of amikacin alone. Therefore, AlgL, which degrades alginate produced by the mucoid strains of *P*. *aeruginosa*, may be used in the treatment of antibiotic-resistant bacteria. Structurally, alginate is a simple, unbranched polysaccharide with a very high molecular weight. It is composed of two uronic acids: β-D-mannuronic acid (M) and its C5 epimer, α-L-guluronic acid (G). *P*. *aeruginosa* alginates are observed as random blocks of MM and MG residues, and not as repeating disaccharides [22, 23]. The alginate produced by CF isolates, which include *P*. *aeruginosa*, is O-acetylated at the C-2 and/or C-3 positions in mannuronic acid residues [24–26].

Acceleration of the alginate degradation process by administration of AlgL has been recognized as a promising pharmacokinetic approach for the treatment of biofilm infections. However, the catalytic activity of wild-type AlgL against alginate and acetylated alginate is not sufficiently high. Therefore, AlgL must be engineered for therapeutic purposes, and a possible mutant with improved catalytic activities against alginate and acetylated alginate needs to be identified. Here we report the first effort towards engineering the protein (enzyme) AlgL for improved activity, based on molecular modeling and rational design. Initially, the *algL* gene was amplified (for mutagenesis) from *Azotobacter vinelandii*, using the plasmid pET28A(+). This combined computational and experimental approach has led to the identification of a number of AlgL mutants exhibiting significantly improved catalytic efficiencies against alginate and acetylated alginate. Leid et al. [27] suggested that the therapeutic use of AlgL in combination with ciprofloxacin or gentamicin is beneficial for combating PA biofilm infection. However, CRPA is not sensitive to ciprofloxacin or gentamicin, but is sensitive to piperacillin/tazobactam (PT) and ceftazidime. Previously, we reported the preventive effect of PT coated silicone against CRPA biofilms [28]. So far, to the best of our knowledge, there has been no report on antibiofilm activity of either chemical or biological compounds against CRPA biofilm formed silicone. The aim of this study was to increase the catalytic efficiency of AlgL toward alginate by means of site-directed mutagenesis based on computational modeling. Additionally, it examined the synergistic antibiofilm-antimicrobial activity of combined PT and AlgL administration, and compared the efficacy of AlgLw with ALgLm using a silicone biofilm *in vitro*.

## Materials and Methods

### Homology Modeling and Simulation of Molecular Dynamics

In order to explore the binding mode of AlgL with its substrate (alginate), we constructed an AlgL-alginate binding structure through homology modeling prior to performing molecular dynamics (MD) simulations. Homology modeling was performed using the Protein Modeling module of the Discovery Studio software (version 2.5.5; Accelrys, Inc., San Diego, CA). MD simulations were carried out using the Sander module of the Amber 12 software package [29]. The computational procedures used in this study were similar to the protocols employed in our previous studies on molecular modeling of protein structures and protein-ligand binding interactions [30, 31]. In brief, the X-ray crystal structure of *Sphingomonas* AlgL species A1-III (PDB entry code 4F13 of the Y246F mutant, with a resolution of 2.21 Å) [32] was used as a template to model the AlgL-alginate complex. The amino acid sequences of the *Azotobacter vinelandii* alginate lyase (GeneBank # AAC32313.1) and *Pseudomonas aeruginosa* AlgL (PalgL, GeneBank # AAA91127.1; from PL-7 subfamily) were directly extracted from the NCBI protein database. Sequence alignment was generated for all amino acid sequences using ClusterW with the Blosum scoring function [33, 34]. The best alignment was selected based on the alignment score and the reciprocal positions of conserved residues in all three proteins, particularly those of the conserved residues on the binding interface (as revealed in the template structure). The coordinates of the conserved residues of all three proteins were directly copied from the template. The coordinates of non-conserved residues were generated with the least geometrical variations using the Protein Modeling module of the program. The substrate alginate, comprising of a minimum length of 4 β-D-mannuronic acid (M) units (MMMM), was initially placed at a site similar to that of the special substrate for the template ALYIII. The geometry of the substrate molecule was optimized by *ab initio* electronic structure calculation at the HF/6-31G* level, using the Gaussian03 program [35]. The HF/6-31G* calculation was also performed to determine the restrained electrostatic potential (RESP)-fitted charges for the substrate molecule. Following construction of the initial AlgL-alginate binding structure, all ionizable residues were set to their standard protonated or deprotonated states. Subsequently, the entire structure was solvated in an orthorhombic box comprising of TIP3P water molecules, keeping a minimal distance of 10 Å between the protein and the boundary of the box. The whole system was neutralized by the addition of 6 sodium counter ions, until a size of 78 Å × 90 Å × 77 Å was achieved. Following the set-up of the whole system, a series of energy minimizations were performed using a conjugate gradient energy-minimization method in the Sander module of the Amber 12 program [29], with a non-bonded cutoff of 10 Å. Finally, the entire system was energy-minimized to obtain a convergence criterion of 0.001 kcal/(mol Å).

In order to further relax the energy-minimized structure of the AlgL-alginate complex, MD simulations were performed using the Sander module of the Amber 12 program [29]. In particular, the Amber ff03 force field [36] was used for all amino acid residues of AlgL and the general Amber force field (gaff) [37] was used for the substrate alginate. MD simulations (0.5 ns) were initially performed on water molecules and counter ions using the NTV ensemble (at T = 300 K), in order to better solvate and equilibrate the complex. Energy minimization was repeated for the entire system, and the same convergence criterion was obtained. The whole system was gradually heated to 300K using a weak-coupling method [38] and equilibrated for 1.0 ns. A 10 Å non-bonded interaction cutoff was utilized throughout the MD simulations, and the non-bonded list was updated at every 25 steps. The particle mesh Ewald (PME) method [39] was applied to treat long-range electrostatic interactions. The lengths of covalent bonds involving hydrogen atoms were fixed using the SHAKE algorithm [40], enabling the use of a 2 fs time step to numerically integrate the equations of motion. Finally, the production MD was allowed to run for approximately 10 ns with a periodic boundary condition (PBC) at a NTP (constant temperature and pressure) of T = 300 K (maintained using Berendsen temperature coupling) and P = 1 atm (using anisotropic molecule-based scaling).

### Experimental Materials

Sodium dodecyl sulfate (SDS), alginate (A7003), phenylmethylsulfonylfluoride (PMSF), imidazole, and dithiothreitol (DTT) were obtained from Sigma Aldrich (St. Louis, MO), whereas cloned *pfu* DNA polymerase was purchased from Stratagene (La Jolla, CA). *Dpn*I endonuclease, protein A-horseradish peroxidase (HRP), polyvinylidene fluoride, and pET28A(+) expression vector were obtained from New England BioLabs (Ipswich, MA), BD Transduction Laboratories (BD Biosciences, Franklin Lakes, NJ), EMD Millipore (Billerica, MA), and Invitrogen (Waltham, MA), respectively. The QIAprep Spin Plasmid Miniprep Kit and Ni-nitrilotriacetic acid (NTA) agarose beads were purchased from Qiagen (Venlo, Netherlands). The ECL Plus Western blotting detection system RPN 2132 was obtained from Amersham Life Science (GE Healthcare, Cleveland, OH). The oligonucleotide primers were synthesized by Bioneer (Daejeon, Korea) and the analysis facility of Chosun University. Acetylated alginate was purified from *Pseudomonas* as previously described, using an alginate-overproducer, *P*. *alkylphenolia* E1 (pAlgG). Purified acetylated alginate was converted to a Na-form by treating with 50 mM ethylene diamine tetraacetic acid (EDTA), and dialysis using 0.5 M NaCl and double distilled water [41].

### Site-directed mutagenesis

All site-directed mutagenesis on AlgL cDNA (GeneBank # AAC32313.1) were performed using the QuikChange site-directed mutagenesis method [42]. Ten primer pairs were used for the polymerase chain reaction (PCR). The sequences of the oligonucleotides used for mutagenesis were: for the mutagenesis of Lys-194 to Ala-194 (K194A), 5′-AGC GAC CTG CCG CTC GCT CGG ATC AAC AAC CAC-3′ and 5′-GTG GTT GTT GAT CCG AGC GAG CGG CAG GTC GCT-3′; for Lys-194 to Glu-194 (K194E), 5′-AGC GAC CTG CCG CTC GAA CGG ATC AAC AAC CAC-3′ and 5′-GTG GTT GTT GAT CCG TTC GAG CGG CAG GTC GCT-3′; for Asn-198 to Ala-198 (N198A), 5′-CTC AAG CGG ATC AAC GCG CAC TCC TAC TGG GCG-3′ and 5′-CGC CCA GTA GGA GTG CGC GTT GAT CCG CTT GAG-3′; for His-199 to Ala-199 (H199A), 5′-AAG CGG ATC AAC AAC GCC TCC TAC TGG GCG GCC-3′ and 5′-GGC CGC CCA GTA GGA GGC GTT GTT GAT CCG CTT-3′; for Lys-245 to Asp-245 (K245D), 5′-CTG CCC AAC GAA CTC GAC CGC CGC CAA CGT GCG-3′ and 5′-CGC ACG TTG GCG GCG GTC GAG TTC GTT GGG CAG-3′; for Arg-246 to Ala-246 (R246A), 5′-AAG GCC TTC CGC ACC GCT ACC GCG CCG ATC ACC-3′ and 5′-GGT GAT CGG CGC GGT AGC GGT GCG GAA GGC CTT-3′; for Tyr-253 to Ala-253 (Y253A), 5′-CAA CGT GCG CTG GCC GCC CAC AAC TAC AGC TTG-3′ and 5′-CAA GCT GTA GTT GTG GGC GGC CAG CGC ACG TTG-3′; for Tyr-253 to Phe-253 (Y253F), 5′-CAA CGT GCG CTG GCC TTC CAC AAC TAC AGC TTG-3′ and 5′-CAA GCT GTA GTT GTG GAA GGC CAG CGC ACG TTG-3′; for Glu-312 to Asp-312 (E312D), 5′-GAG GAT CAG GAC ATG GAT GAT CTG GAG ACC GAC-3′ and 5′-GTC GGT CTC CAG ATC ATC CAT GTC CTG ATC CTC-3′; and for Lys-319 to Ala-319 (K319A), 5′-CTG GAG ACC GAC GCC GCA TTC TCC TGG CTG GAA-3′ and 5′-TTC CAG CCA GGA GAA TGC GGC GTC GGT CTC CAG-3′. In these sequences, the underlined bases indicate the mutations in the bases. Double mutants K194E-K245D and K245D-K319A were prepared using the template cDNA of K245D, and the above-mentioned K194E and K319A primers. Triple mutants K194E-K245D-E312D and K194E-K245D-K319A were prepared using the template cDNA of the K194E-K245D mutant and the E312D or K319A primers. The PCR process was carried out using *Pfu* DNA polymerase. The PCR product was treated with *Dpn*I endonuclease to digest the parental DNA template. The mutant DNA sequences were confirmed by DNA sequencing.

### Expression and purification of AlgL and its mutants

A recombinant plasmid was used for the transformation of *Escherichia coli* BL-21(DE_3_) cells. The cells were grown in 500 mL Luria-Bertani (LB) medium supplemented with 100 μg/mL kanamycin, at 37°C in a shaker incubator (250 rpm) until an OD_600_ of 0.6 was achieved. At this OD, isopropyl β-D-1-thiogalactopyranoside (IPTG) was added to a final concentration of 1 mM, and the cells were allowed to grow for an additional 12 h at 20°C. The cells were then harvested by centrifugation at 6000 × *g* for 15 min at 4°C. The cell pellet was resuspended in 20 mL cold cell lysis buffer [50 mM Tris, 300 mM NaCl, 5 mM imidazole buffer (pH 8.0), containing 1 mM PMSF and 1 mM DTT]. The cells were ruptured by sonication. The cell lysate was cleared by centrifugation at 10,000 × *g* for 20 min. The extract was slowly loaded onto the Ni-NTA agarose bead column, which was equilibrated at 4°C with a column lysis buffer [50 mM Tris, 300 mM NaCl, 5 mM imidazole buffer (pH 8.0), containing 1 mM PMSF and 1 mM DTT]. The column was washed using a column buffer [50 mM Tris, 500 mM NaCl, 20 mM imidazole buffer (pH 8.0)] until an OD_280_ of below 0.005 was achieved. The AlgL was then eluted from the Ni-NTA agarose bead column by incubation with the elution buffer [50 mM Tris, 300 mM NaCl, 300 mM imidazole buffer (pH 8.0) containing 1 mM PMSF and 1 mM DTT] at room temperature for 5 min. The concentration of the purified enzyme was determined, and its purity was assessed by sodium dodecyl sulfate polyacrylamide gel electrophoresis (SDS/PAGE).

### AlgL assay

All biochemical characterization assays were performed using the purified enzyme resuspended in 50 mM Tris-HCl buffer (pH 7.5). An aliquot (0.2 mL) of the enzyme solution was added to 2.8 mL of previously incubated substrate solution (at 37°C), comprised of 0.2% sodium alginate dissolved in 50 mM Tris-HCl buffer (pH 7.5). The enzymatic reaction was terminated by heating the mixture in boiling water for 5 min. Lyase activity was quantitatively measured using the thiobarbituric acid method [43]. One unit of the enzyme activity was defined as 1 μmol liberated product per min.

### Biofilm formation and treatment *in vitro*

Silicone sheets (0.5 cm × 0.5 cm) were prepared in 16-well plates. Each isolate was grown to the logarithmic phase in tryptone soy broth (TSB) at 37°C for 24 h, harvested by centrifugation, resuspended in a volume of TSB, and monitored spectrophotometrically to yield approximately 5 × 10^6^ colony-forming units (CFU) per milliliter. Before each experiment, several colonies were used to inoculate 5 mL TSB at 37°C for 24 h in ambient air. Subsequently, 100 μL of this culture was used to inoculate 50 mL of medium, from which biofilms were grown on silicon introduced by suspension from a stainless steel hook. After a total incubation period of 2 days, the silicone sheets were collected, dipped once in sterile 0.9% NaCl, and gently dried by a minimal touch with a soft tissue to wash off planktonic microorganisms as well as any remaining TSB. After separation of the CRPA biofilm-formed silastic sheets, they were transferred to six new sets of 12-well plates with TSB. Six different treatments were performed: control (no treatment; Group I), 1 mL PT (Tazocin, 4%) with 1 mL phosphate-buffered saline (Group II), 1 mL PT (Tazocin, 4%) with 1 mL wild-type AlgL (Group III), 1 mL PT (Tazocin 4%) with 1 mg mutant K197D/K321A AlgL (Group IV), 1 mg wild-type alone (Group V), and 1 mL mutant K197D/K321A alone (Group VI). All groups were incubated for 1 d. All experiments were carried out in triplicate, and the mean of results was reported.

### Quantitative assessment of biofilm development

Each of the four silicone sheets in the six different treatment groups was carefully washed five times with sterile phosphate-buffered saline (PBS) to remove non-adherent bacteria. Silicone sheets were transferred into 15-mL conical tubes containing 5 mL of PBS. The tubes were vigorously vortexed for 2 min to free the bacteria attached to the surface of each silicone sheet and sonicated at low power (45 kHz) for 3 min (Microson Ultrasonic Cell Disruptor XL, Misonix Inc., NY, USA) to disperse the bacterial cells. This regimen has been found to remove all adherent bacteria without affecting viability [44]. Samples of 200 mL from the sonicated biofilms were serially diluted in 0.9% saline solution and were plated onto TSB to determine the total number of viable cells. The plates were incubated for 24 h at 37°C and the colonies were counted manually. Statistical differences between independent groups were assessed using one-way analysis of variance (ANOVA). A Mann-Whitney U test was performed to compare between two groups. Statistical significance was defined as *p* < 0.05.

### Evaluation of the silicone sheet surface

Each of the four silicone sheets from four different treatment groups (I, II, III, and IV) was immersed overnight in fresh 2% glutaraldehyde and post-fixed by O_S_O_4_ solution. Groups E and F were not included because of the absence of antibiofilm effect. After the dehydration process, the films were prepared by critical point drying and platinum sputter coating. All the prepared specimens were investigated for 30 min each to determine CRPA biofilm formation on the surface of the silicon sheet. The surface of the CRPA biofilm on each silicone sheet was examined by scanning electron microscopy (SEM, FE-SEM, Hitachi, Tokyo, Japan), atomic force microscopy (AFM, XE-100, Park System co., Suwon, Korea) and the surface roughness of three different groups II, III, and IV was determined by a nanosurface 3D optical profiler (NV-E1000; Nano System Co Ltd).

### Antibiofilm test by live/dead staining

Each of the four silicone sheets from four different treatment groups (I, II, III, and IV) intended for imaging was subjected to a LIVE/DEAD BacLight^™^ Bacterial Viability Kit (ThermoFisher Scientific Co., Waltham, MA, USA) and staining kits for CRPA. In this study, we omitted the groups E and F, because SEM and AFM showed no effect of AlgL-only treatment groups. Directly after the silicone sheets were removed, they were stained for 15 min in the dark at room temperature with both the live/dead viability stains containing SYTO 9 dye (3.34 mM) and propidium iodide (20 mM). A series of about 20 images was generated for each biofilm on the silicone sheets using confocal laser scanning microscopy (laser scanning confocal microscope, TCS SP5/AOBS/Tandem, Leica, Germany).

## Results and Discussion

### AlgL structure and AlgL-alginate binding

**Fig 1:**
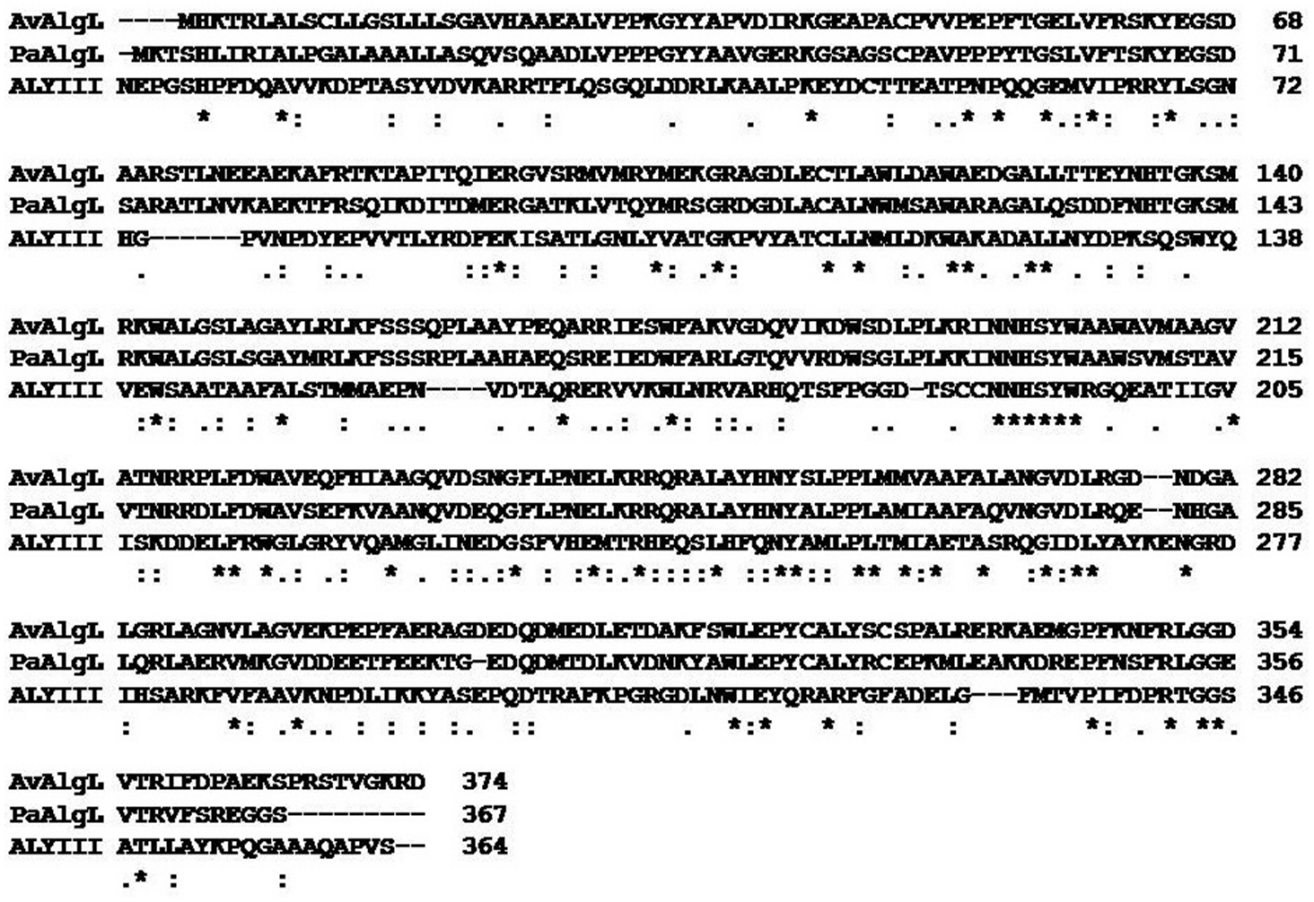
Sequence alignment of *Azotobacter vinelandii* alginate lyase (AvAlgL) with *Pseudomonas aeruginosa* alginate lyase (PaAlgL), and with the template *Sphingomonas* alginate lyase species A1-III (ALYIII). Stars, colons, and semicolons represent the conserved residues among these three proteins, revealing an overall homology of 55.3% among these three proteins.

**Fig 2:**
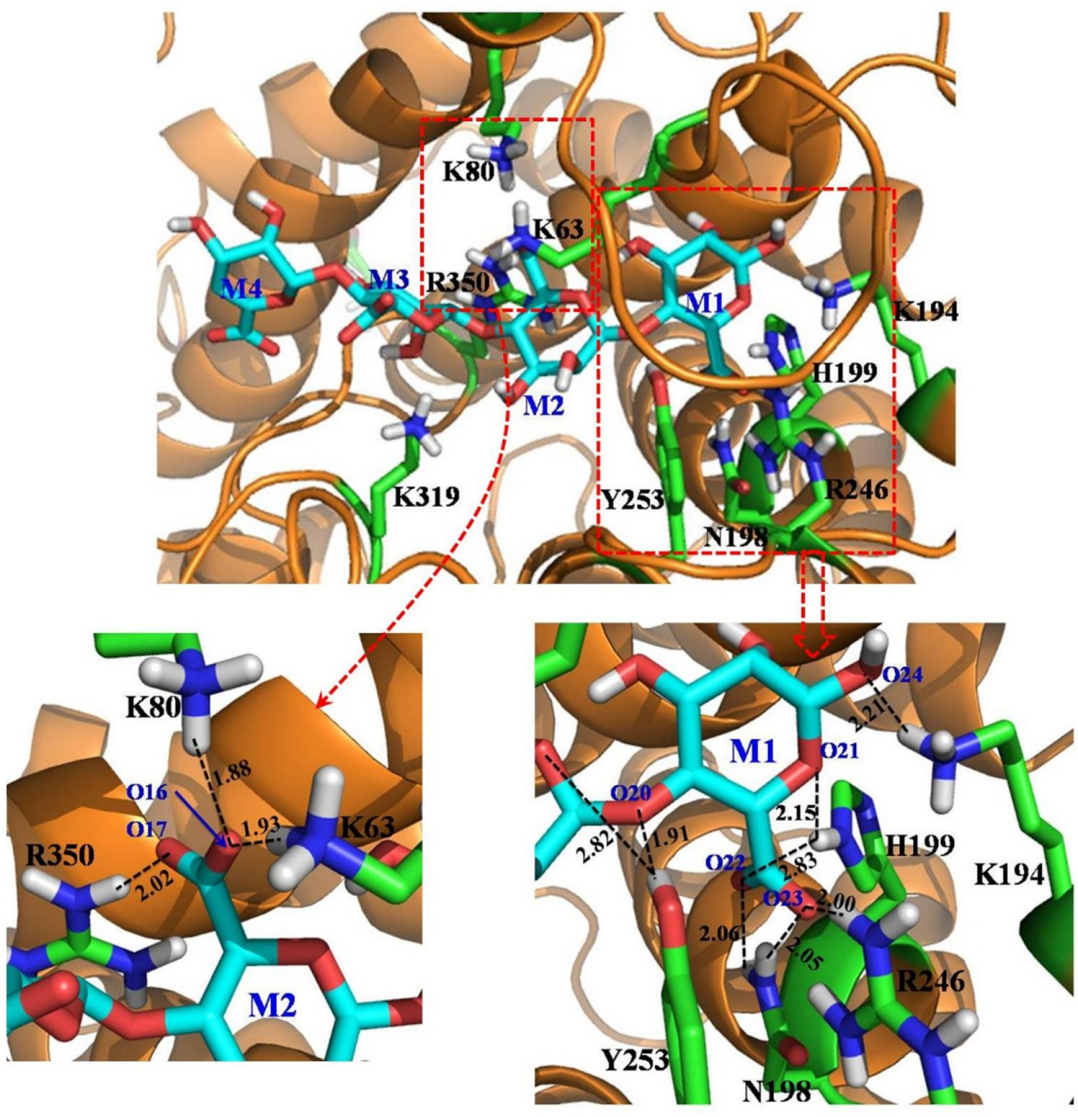
MD-simulated structure of the AlgL-alginate complex. The top panel: AlgL is represented as a ribbon in golden color, and the substrate alginate is shown in the stick style with four units labeled as M1 to 4. Residues within 5 Å around the substrate are represented in the stick style and colored according to atom type. The bottom-right panel: the atomic details of intermolecular interactions on the binding interface of AlgL and the M1 unit of the substrate alginate. The atom names of M1 are labeled, and the intermolecular hydrogen bonds are represented as dashed lines with distances labeled (in Å units). The bottom-left panel: local intermolecular interactions between AlgL and the M2 unit of the substrate alginate are shown.

Fig 1 depicts the aligned sequences between the template ALYIII and two members of the sub-family PL-7 (AlgL and PalgL). An overall high homology (55.3%) among the three proteins suggested high conservation of the residues at the substrate binding cleft (e.g. N198, H199, and Y253). The binding structure of the AlgL-alginate complex, derived from the trajectory of 10 ns MD simulation is depicted in Fig 2. Compared to the X-ray crystal structure of the template (PDB code 4F13) [32], the geometrical root-mean square deviation (RMSD) for the Cα atoms of the modeled AlgL structure was very low (1.03 Å), suggesting the high fidelity of the modeled structure. As seen in Fig 2, the substrate alginate is located at a similar cleft, as observed in the X-ray crystal structure of the template ALYIII. According to the X-ray crystal structure of ALYIII [32], the loop (formed by residues 64 to 85; ALYIII numbering) was induced to move to the binding cleft and cover the substrate binding site. The corresponding loop (created out of residues 57 to 78) of AlgL is located immediately above the bound substrate alginate (Fig 2). For convenience, the entire substrate consists of four structural units (M1 to 4), as indicated in Fig 2. The substrate unit M1 is closely packed with the side chains of the residues K194, N198, H199, R246, and Y253, through extensive electrostatic and hydrogen-bonding interactions. In particular, the substrate M1 unit formed strong hydrogen-bonding interactions with the -NH_2_ group on the N198 and H199 side chains, the positively charged group on the R246 side chain, and the hydroxyl hydrogen atom on the Y253 side chain. The negatively charged oxygen atoms in the carbonyl group of the M1 unit of the substrate also interact with the positively charged group of the R246 side chain via electrostatic interactions. The hydroxyl group present in the M1 unit of the substrate is connected via a hydrogen bond with the positively charged head group in the K194 side chain of AlgL. The Y253 residue forms a strong hydrogen bond with the oxygen atom connecting the M1 and M2 units of the substrate, which may initiate the catalytic reaction of substrate degradation. The M2 unit of the substrate alginate primarily interacted with several positively charged residues of AlgL, such as K63, K80, and R350 (the bottom-right panel of Fig 2), through hydrogen bonds and electrostatic interactions. The carbonyl group of the M3 unit of the substrate is close to the positively charged head group of the K319 residue of AlgL, whereas the M4 unit of the substrate is completely exposed to the surrounding solvent water molecules. The AlgL-alginate binding mode was stable during the long-term MD simulation (see S1 to S5 Figs).

Based on the modeled AlgL-alginate binding structure, various amino-acid residues were determined to be crucial for binding with the substrate and, consequently, for enzyme activities against the two substrates. Based on the modeled structure depicted in Fig 2, the N198, H199, and R246 residues were packed together with the carbonyl group of the M1 unit of the substrate via strong electrostatic and hydrogen-bonding interactions, suggesting that these three residues are critical for the M1 orientation in the binding cleft. The residue Y253 showed double hydrogen bonding interactions with the substrate alginate and the Y64 residue of AlgL (see Fig 2, the black curve in S3 Fig, and the cyan curve in S5 Fig), indicating that any mutation affecting Y253 could probably inactivate the enzyme. The results of site-directed mutagenesis (see Table 1) showed that the N198A, H199A, R246A, Y253A, and Y253F mutations indeed resulted in the enzyme inactivation, which is consistent with the mode of the AlgL-alginate binding described above. Based on the simulation, the residue K194 appeared to show a small percentage of hydrogen bonding with the hydroxyl group at the M1 subunit of the substrate (see the blue curve in S3 Fig). As observed in the binding structure, this residue is located near the positively charged R246 side chain (see the bottom-right panel of Fig 2), indicating the limited role of K194 in substrate binding. This point is supported by the observation from site-directed mutagenesis showing that K194A and K194E mutations have a very small effect on the enzyme-substrate binding affinity (see the *K*_m_ values listed in Table 1). According to the modeled structure depicted in Fig 2, the residue K319 did not form stable hydrogen bonds with the M3 unit of the substrate alginate; consequently, its contribution to the enzyme-substrate binding affinity should be insignificant, which was confirmed by our experimental observation that the K319A mutation did not significantly change the *K*_m_ values (Table 1). The K245 residue did not have any direct contact with the bound substrate; therefore, the K245D mutation should have no significant effect on substrate binding (Table 1), apart from a possible change in charge distribution on the enzyme surface. Interestingly, the E312D mutation significantly increased the enzyme-substrate binding affinity, as seen in the *K*_m_ values in Table 1. According to the modeled enzyme-substrate binding structure, residue E312 is located in the same loop as that of K319 (Fig 2). A subtle change, such as the E312D mutation, might significantly affect the possible intra-molecular interactions (e.g. local intra-molecular interactions around E312 and K319). Consequently, this could result in a direct change in the inter-molecular interactions of the loop containing the E312 and K319 residues with the M3 and M4 units of the substrate alginate. We recommend that the exact role of the E312 residue in substrate binding be explored further in future research. Further observation of the modeled AlgL-alginate binding structure also suggested that the substrate binding cleft and the adjacent molecular surface of the enzyme are covered with positively charged residues (e.g. K194, K245, R246, and K319). These data suggest that long-range electrostatic interactions between the positively charged residues and the substrate would not assist in dissociation of the substrate and the products of the catalytic reaction. A reversal in the charges and/or a decrease in the positive charges of some of these residues (e.g. K194E) might facilitate dissociation of the products from the catalytic reaction. This is in agreement with the observed increase in *k*_cat_ resulting from the mutations such as K194A, K194E, K245D, K319A, and the combination of these mutations (Table 1).

**Table 1:** *K*_m_, *k*_cat_, and *k*_cat_*/K*_m_ values of mutants.

Mutants	*k*_cat_ (min^-1^)		*K*_m_ (mg∙mL^-1^)		*k*_cat_/*K*_m_ (mL∙min^-1^∙mg^-1^)		Relative activity	
	Sodium alginate	Acetylated alginate	Sodium alginate	Acetylated alginate	Sodium alginate	Acetylated alginate	Sodium alginate	Acetylated alginate
Wild type	63.8 ± 4	85.9 ± 10	0.156 ± 0.02	0.468 ± 0.03	409.0	183.6	1.0	1.0
K194A	104.6 ± 9	190.2 ± 19	0.119 ± 0.01	0.458 ± 0.02	880.4	415.3	2.2	2.3
K194E	149.9 ± 11	310.7 ± 22	0.159 ± 0.02	0.349 ± 0.02	942.9	890.4	2.3	4.9
N198A	ND	ND	ND	ND	ND	ND	-	-
H199A	ND	ND	ND	ND	ND	ND	-	-
K245D	137.3 ± 28	324.9 ± 51	0.150 ± 0.02	0.652 ± 0.03	916.2	498.2	2.2	2.7
R246A	ND	ND	ND	ND	ND	ND	-	-
Y253A	ND	ND	ND	ND	ND	ND	-	-
Y253F	ND	ND	ND	ND	ND	ND	-	-
E312D	69.6 ± 7	152.5 ± 12	0.094 ± 0.01	0.391 ± 0.02	744.1	389.4	1.8	2.2
K319A	64.1 ± 3	165.1 ± 14	0.126 ± 0.01	0.396 ± 0.01	508.4	416.6	1.2	2.3
K194E/K245D	256.3 ± 26	504.3 ± 38	0.153 ± 0.02	0.625 ± 0.05	1673.0	806.4	4.1	4.4
K245D/K319A	197.9 ± 13	352.9 ± 52	0.134 ± 0.01	0.308 ± 0.03	1476.6	1145.5	3.6	6.2
K194E/K245D/E312D	282.3 ± 35	839.8 ± 65	0.095 ± 0.01	0.955 ± 0.07	2950.2	879.1	7.2	4.8
K194E/K245D/K319A	280.9 ± 21	901.5 ± 78	0.103 ± 0.02	0.768 ± 0.06	2718.9	1173.7	6.7	6.4

ND, not-detectable activity

### Identification of catalytic residues of AlgL

**Fig 3:**
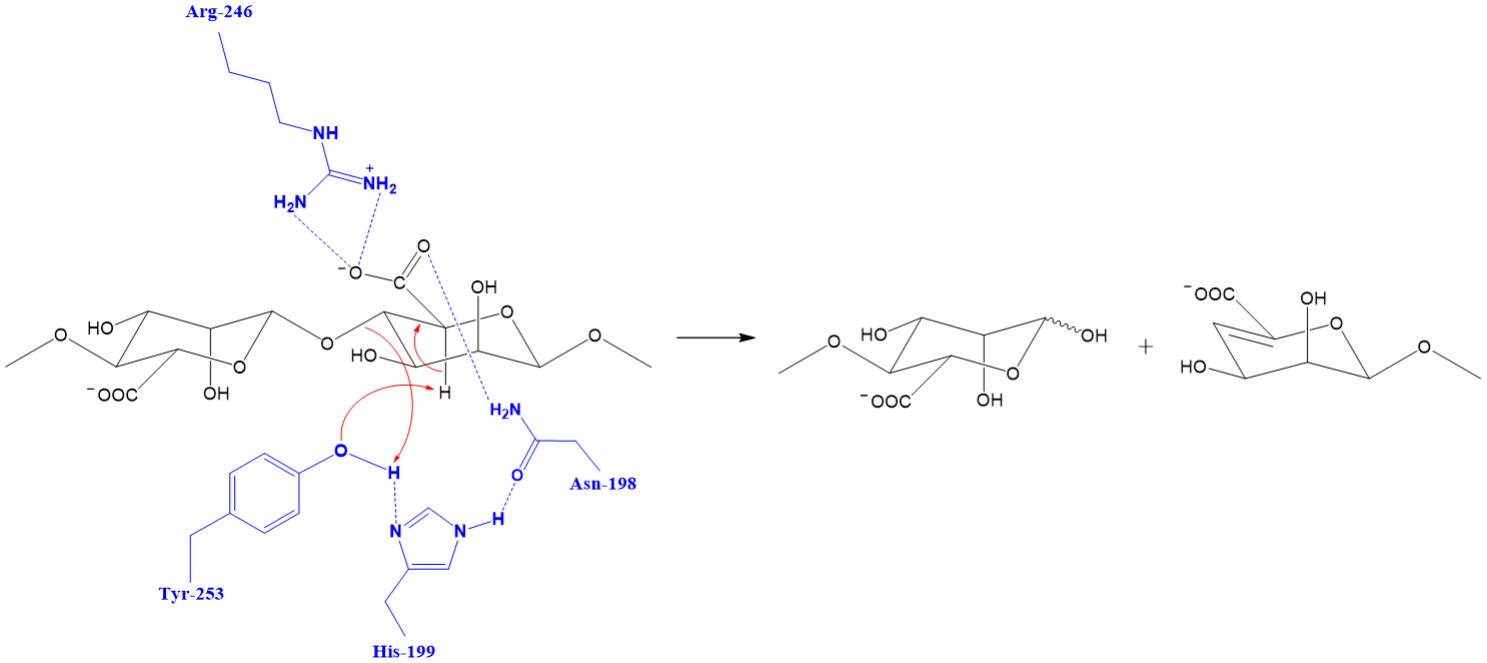
Schematic view of the β-elimination reaction mechanism.

AlgL, isolated from *Azotobacter vinelandii*, is a member of the polysaccharide lyase family-7 that catalyzes the depolymerization of polyuronides via β-elimination [45]. Although the crystal structure of this AlgL is yet to be determined, that of A1-III from *Sphingomonas* species A1 has been previously elucidated [32]. Furthermore, the crystal structure of the binary complex (including the enzyme and tetrasaccharide substrate) of A1-III has also been characterized [46]. An examination of the high-resolution crystal structure of the binary complex, the 3D model of AlgL, and our experimental data led to the development of a possible catalytic mechanism for AlgL [32, 46]. The “Asn-His-Tyr catalytic triad” is presently considered important in polysaccharide lyase-mediated catalysis. Our experimental results indicated that R246 also plays an important role in the catalytic degradation of substrates (Fig 3). Catalytically important residues of AlgL were identified via site-directed mutagenesis to include N198, H199, R246, and Y253; inactivating mutations in any of these residues resulted in almost complete loss of AlgL activity towards the two substrates (alginate from brown algae and acetylated alginate from *Pseudomonas*) (Table 1). The 3D model for the binary complex of AlgL-alginate depicted in Fig 2 shows neutralization of the C5 carboxylate group of alginate as a result of the hydrogen-bonding with the protonated amine group of R246. In addition, the amine group on the side chain of N198 forms a hydrogen bond with an oxygen atom of the C5 carboxylate group and, thus, fix the C5 carboxylate moiety. The 3D model of the binary complex of AlgL-alginate revealed that the residues H199 and Y253 function as an acid/base pair. Based on this information, the N198, H199, R246, and Y253 residues of AlgL were subjected to site-directed mutagenesis (to alanine or phenylalanine: N198A, H199A, R246A, Y253A, and Y253F; see Table 1). None of the mutants displayed any detectable activity against the substrates. Therefore, the catalytic mechanism of AlgL isolated from *Azotobacter vinelandii* appeared to be dependent on four residues (N198, H199, R246, and Y253), which is consistent with a neutralization-acid/base mechanism.

### Improving AlgL activity by site-directed mutagenesis

**Fig 4:**
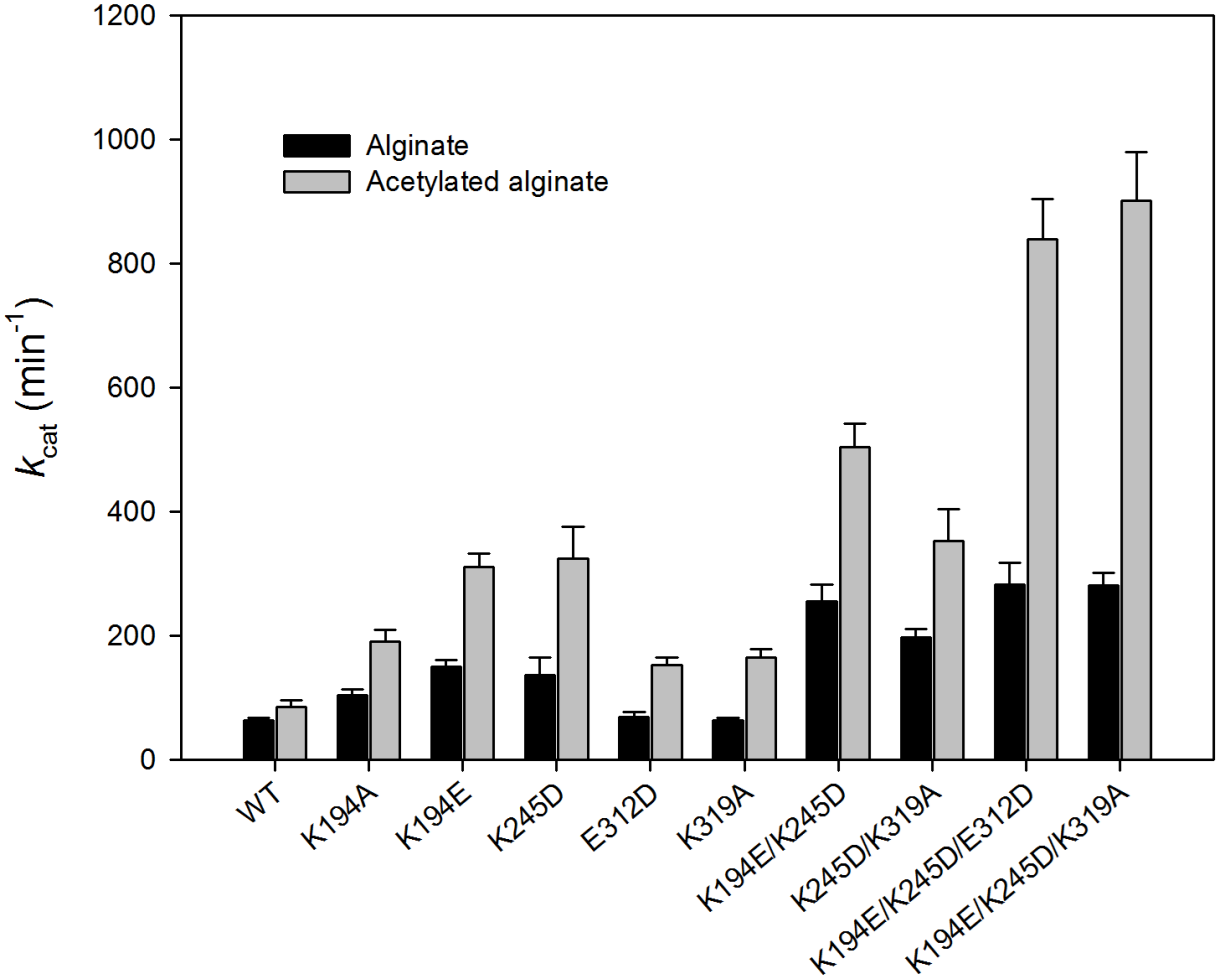
Comparison of enzyme activities of wild type AlgL and mutant AlgL K194A, K194E, K245D, E312D, K319A, K194E/K245D, K245D/K319A, K194E/K245D/E312D, and K194E/K245D/E312D against two substrates (alginate from brown algae and acetylated alginate from *Pseudomonas*). The purified enzymes were assayed for AlgL activity. AlgL activity was determined by the thiobarbituric acid method, as described in the “**Experimental Procedures**” section.

Based on the computational analysis, site-directed mutagenesis of the K194, K245, E312, and K319 residues of AlgL to alanine, glutamic acid, and aspartic acid (K194A, K194E, K245D, E312D, and K319A) was conducted. The mutants has an improved catalytic efficiency (*k*_cat_/*K*_m_) against the two substrates by nearly 2-fold, with the exception of K194E (with acts against the acetylated alginate substrate), which has a 5-fold higher catalytic activity compared to the wild-type enzyme (Table 1 and Fig 4). Double and triple mutants (K194E/K245D, K245D/K319A, K194E/K245D/E312D, and K194E/K245D/K319A) were also prepared from the single-site mutants K194A, K194E, K245D, E312D, and K319A. The *k*_cat_/*K*_m_ ratios of the double mutants K194E/K245D and K245D/K319A against the alginate substrate showed nearly 4-fold increase, compared to the wild-type. The *k*_cat_/*K*_m_ ratio of the double mutant K245D/K319A against the acetylated alginate substrate was increased by 6-fold, as shown in Table 1. The *k*_cat_/*K*_m_ ratios of the triple mutants K194E/K245D/E312D and K194E/K245D/K319A against acetylated alginate did not change compared to the double mutants. However, the triple mutants were observed to be more active against alginate (activity) compared to the double mutants (Table 1 and Fig 4).

### Synergistic effect of antibiotics and AlgL on biofilm disruption

**Fig 5:**
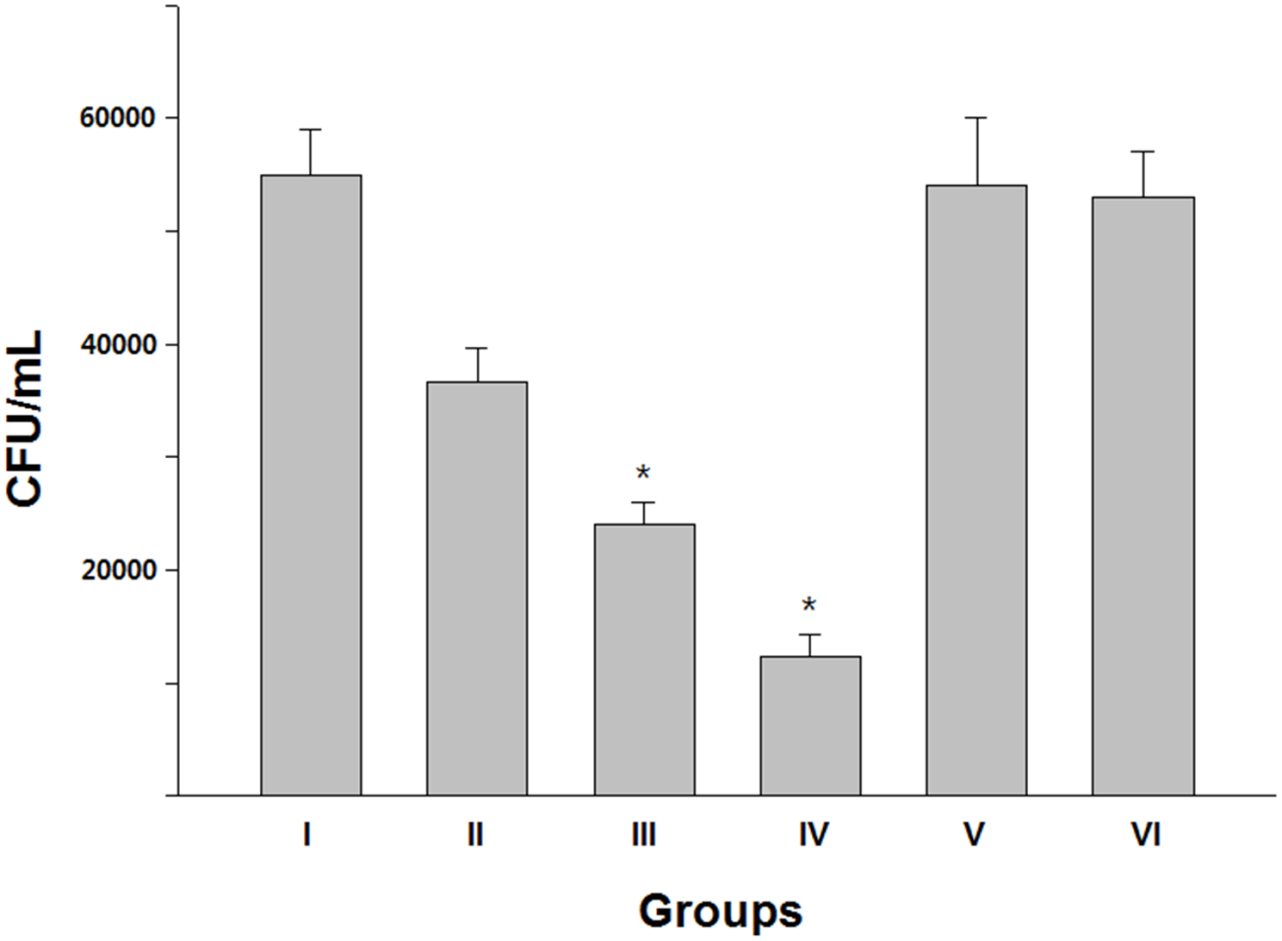
The combination of PT with mutant K194E/K245D/K319A was significantly more effective than PT with wild-type at reducing the CRPA biofilm (*p* < 0.05) and group IV was significantly reduced compared to group III (*p* < 0.05). However, no effect of either type of AlgL alone was detected in reducing the CRPA biofilm.

**Fig 6:**
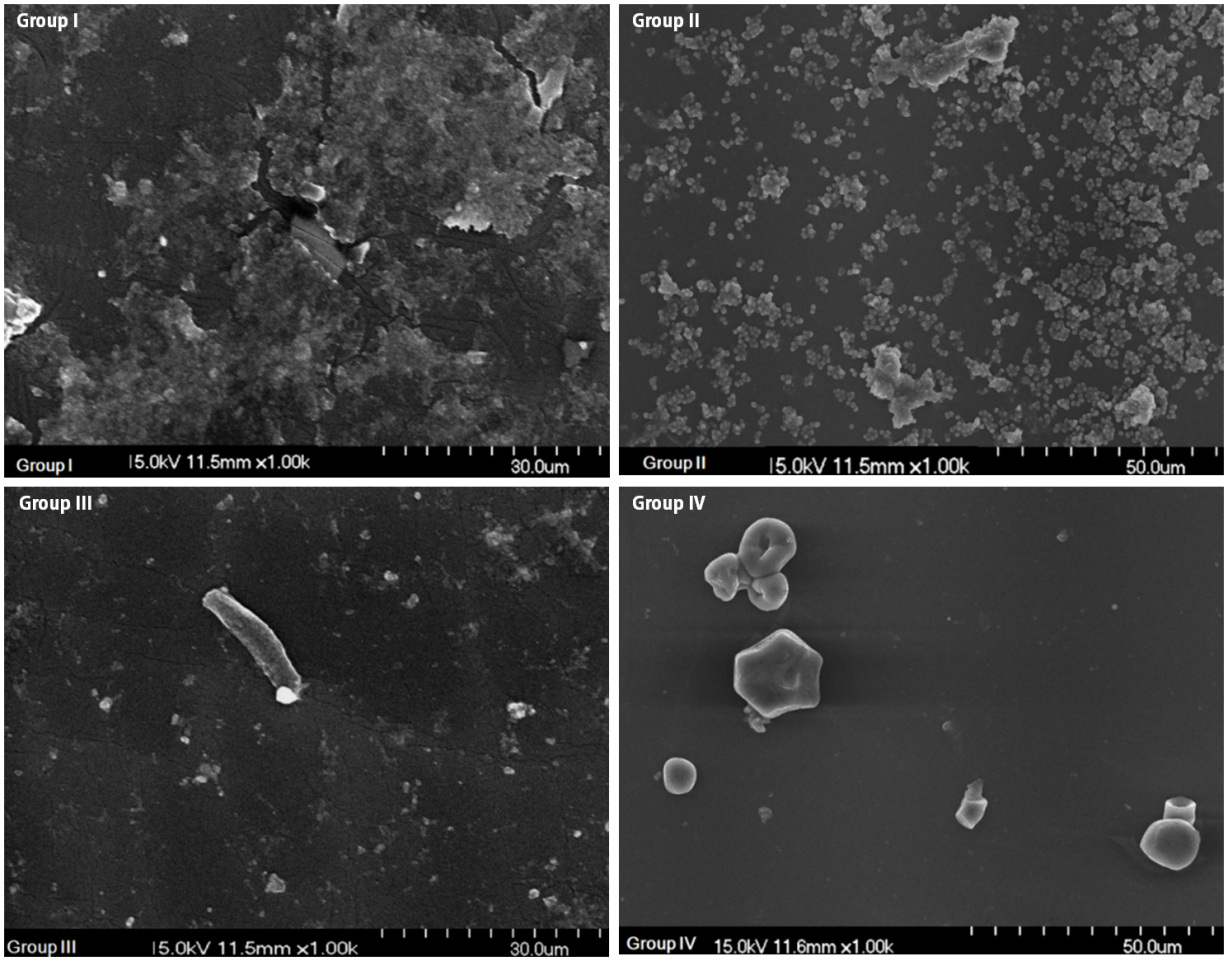
Biofilm formation in each group was identified by scanning electron microscopy. The biofilm disruption effect was best achieved in group IV compared to other groups.

**Fig 7:**
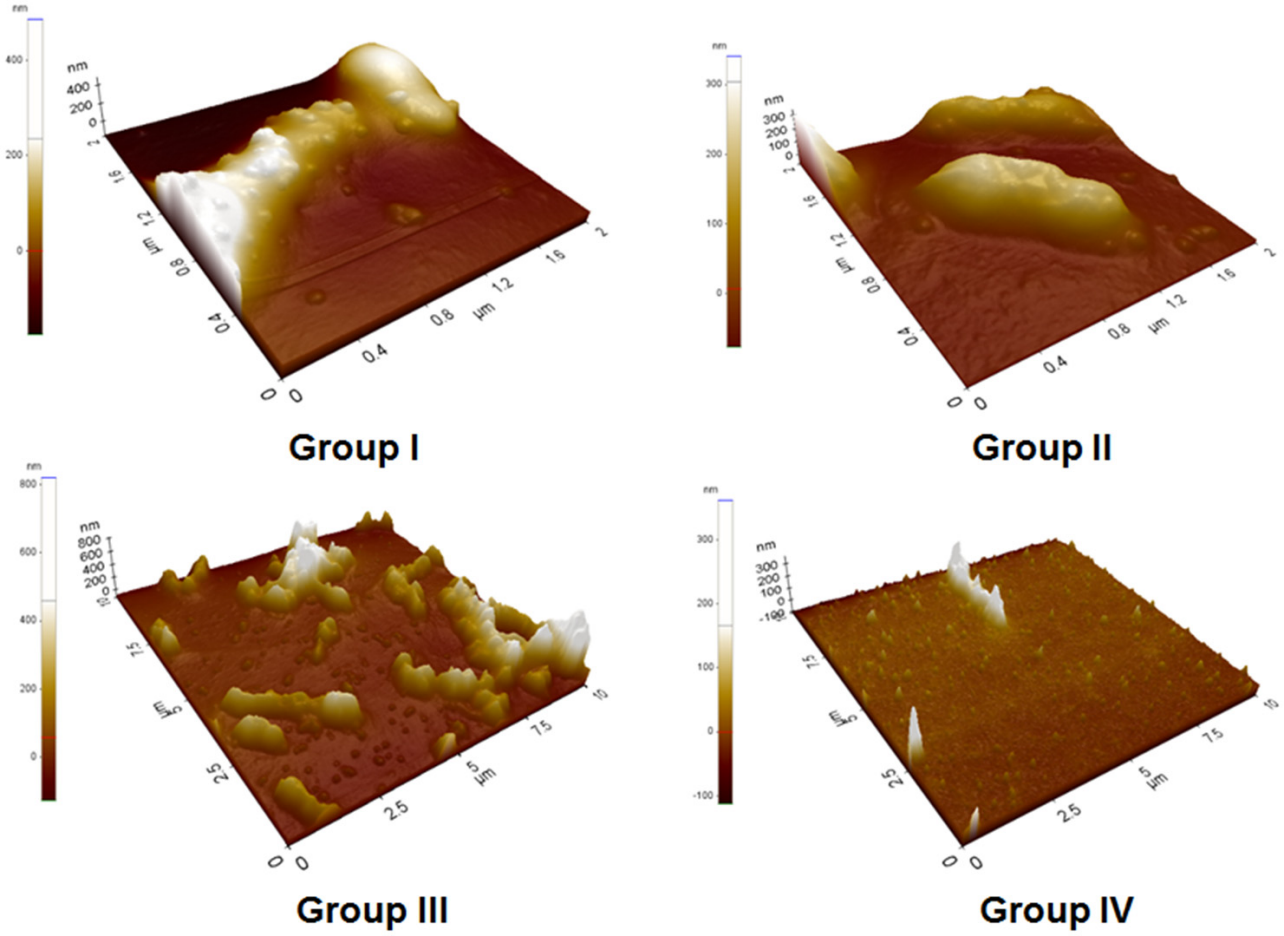
Antibiofilm effect of each group (II, III and IV) compared to the control group by atomic force microscopy. Group IV showed a prominent antibiofilm effect compared to the other two groups.

Depicted in Fig 5 are viable planktonic cells (suspended) of CRPA (log_10_ CFU/mL) after 24 h incubation in silicone sheets, which were treated in five different groups. Colony counts of the controls and AlgL-alone treatment groups were significantly higher than those on the silicone sheet consisting of treatment with PT with or without the wild-type and mutant AlgL (*p* < 0.002). The combined PT treatment along with mutant K194E/K245D/K319A was significantly more effective than PT treatment with wild-type in reducing the CRPA biofilm (p < 0.05). However, no effects of either type of AlgL used alone were detected in reducing the CRPA biofilm. SEM (Fig 6) and AFM (Fig 7) showed a robust CRPA biofilm in the control group. On the other hand, there was reduced CRPA biofilm formation in the silicone sheet with PT combined with the wild-type and the mutant K194E/K245D/K319A. The addition of the mutant K194E/K245D/K319A to PT at a low concentration enhanced its capacity to disrupt the CRPA biofilm as compared to addition of the wild-type. Atomic force microscopy (AFM) is emerging as a potent alternative tool for studying biofilms. This differs from SEM, which works by surface scattering and absorption of electrons and therefore lacks vertical resolution [28].

**Fig 8:**
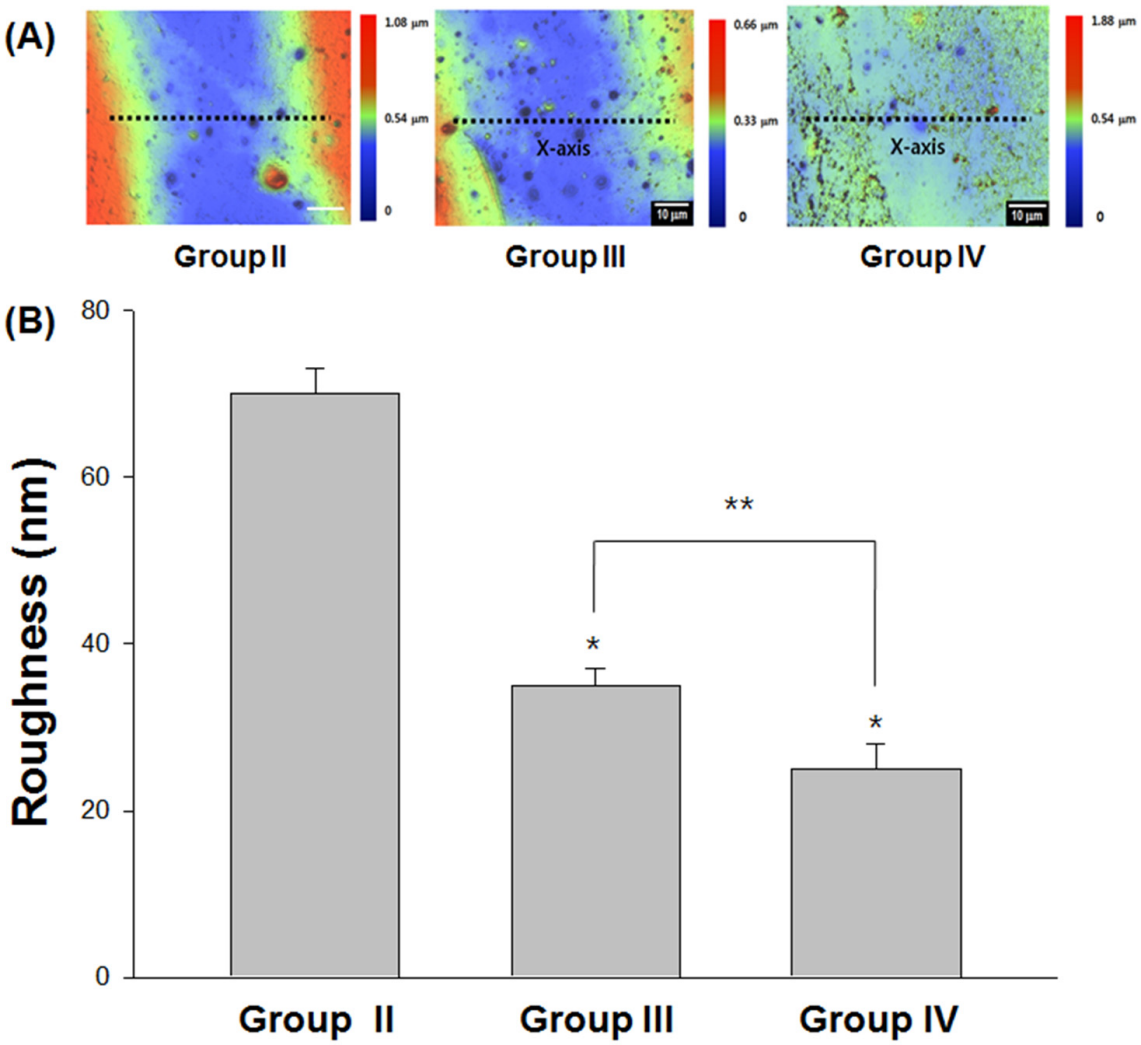
Biofilm thickness expressed by surface roughness measurement using the 3D nanosurface optical profiler. Among three different treated groups, PT with AlgL mutant treated group IV had a significantly lower surface roughness than group III. One-way ANOVA showed statistically significant differences among the mean values of surface roughness of the three groups (*P* < 0.001). Group IV was statistically thinner than group III (*P* < 0.05) using the Mann-Whitney U test.

**Fig 9:**
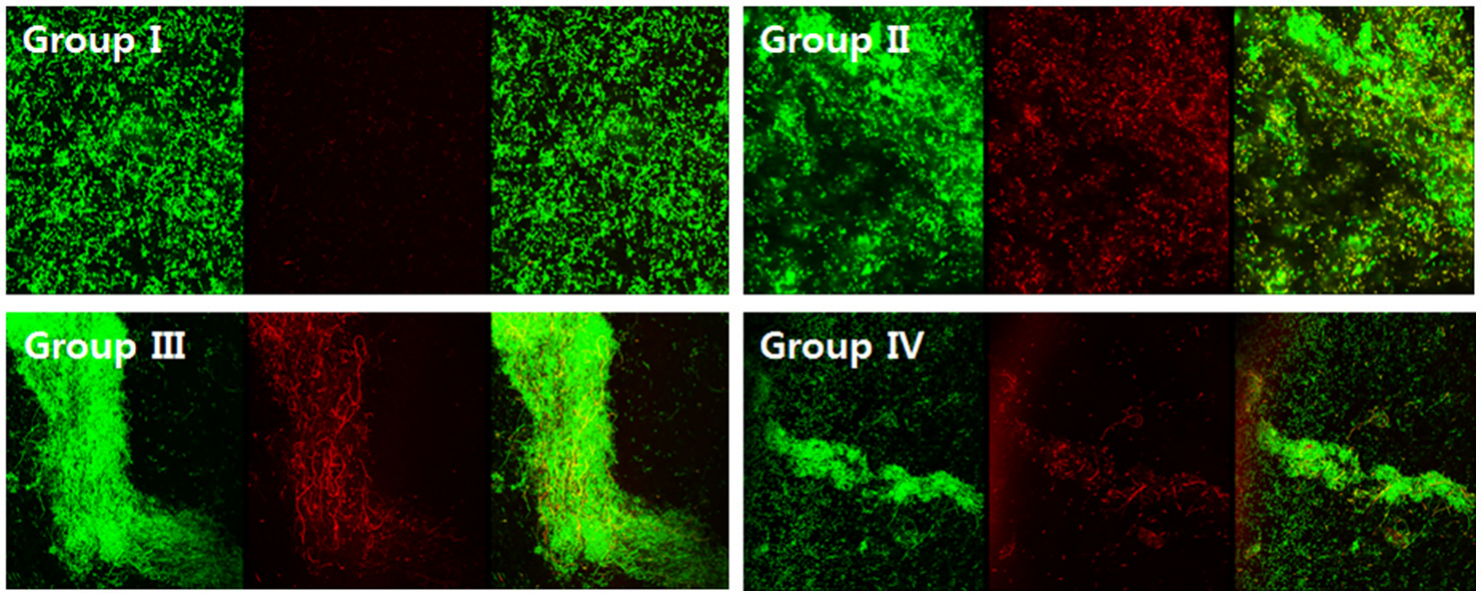
The number of live cells identified in the biofilms of PT with mutant group is less than the other groups.

The surface roughness value determined by nano-surface 3D optical profiler was the highest in group II, PT only treated silastic sheet compared to the other two groups III, IV (Fig 8). PT with AlgL mutant treated group IV had a significantly lower surface roughness than group III. One-way ANOVA showed statistically significant differences among the mean values of surface roughness among the three groups (*P* < 0.001). The live and dead bacteria showed green and red fluorescence, respectively. The control silicone sheet biofilm showed green fluorescence for live cell densities; in contrast, the study group silicone sheet showed robust red fluorescence for dead cell densities. The amount of dead cells in the biofilms of PT with mutant or wild-type groups was determined. Addition of the mutant to PT at low concentrations enhanced its capacity to disrupt the CRPA biofilm as compared to the addition of the wild type (Fig 9). Three global non-microbicidal strategies have been proposed to target pathogenic bacteria with biofilm formation ability by (i) preventing microbial attachment to a surface, (ii) disrupting biofilm development and/or affecting biofilm architecture to enhance the penetration of antimicrobials, and (iii) affecting biofilm maturation and/or inducing its dispersion and degradation [44, 47, 48, 49]. The inability of antimicrobial agents to penetrate into the biofilm network (one of the most important reasons for the development of antibiotic-resistant bacteria) could be overcome through the application of matrix-targeting enzyme treatment [50, 51]. The combination of ciprofloxacin or gentamicin with AlgL has also been tried with PA biofilms. AlgL improved antibiotic activity in reducing the PA biofilm [16–21]. Nowadays, there are increasing incidences of antibiotic-resistant bacteria, such as methicillin-resistant *Staphylococcus aureus* and CRPA. However, there are no reports concerning the effect of AlgL on CRPA biofilms.

In the present study, AlgL combined with PT improved the disruption of CRPA biofilms. Our results are consistent with previous reports [16, 17, 19, 20] revealing enhanced biofilm disruption activity when an antibiotic was combined with AlgL. Further research, including *in vivo* studies, in this area are necessary.

## Conclusion

The crystal structure of A1-III from *Sphingomonas* species A1 was employed to build a 3D model of *Azotobacter vinelandii* AlgL. The secondary structure and the results of a systematic analysis of hydrogen bonding patterns of several AlgL crystal structures were used to refine the catalytic residues of AlgL. This analysis revealed a number of residues and tertiary structure motifs that were functionally conserved across most of the available AlgL templates. Computational simulations, site-directed mutagenesis, and AlgL activity assays allowed a detailed understanding of the interactions between AlgL and its substrate. The experimental data was observed to be consistent with the simulated predictions. A detailed analysis of the modeled AlgL-alginate binding structure revealed that the most important contributions were from positively charged residues (K63, K194, K245, R246, and K319). The catalytic residues of AlgL were also identified via site-directed mutagenesis to be N198, H199, R246, and Y253, and the mutations on these residues resulted in an almost complete loss of the AlgL activity. This is illustrated further in the 3D model of AlgL, which was generated based on the A1-III (from *Sphingomonas* species A1) crystal structures. Therefore, our data suggested that the C5 carboxylate group of the substrate was neutralized by R246 and stabilized further by N198, with H199/Y253 acting as the base and Y253 playing the role of an acid. We have also modified wild-type AlgL by site-directed mutagenesis in order to improve catalytic activities against alginate substrates. The most potent mutant was determined to be K194E/K245D/K319A, which has a 10-fold higher *k*_cat_ value compared to the wild-type enzyme. The results obtained from our *in vitro* antibiofilm studies suggest the potential to use AlgLm for treatment of *in vivo* CRPA biofilm infections. As alginate plays a role in preventing the killing of *Pseudomonas aeruginosa* biofilms, AlgL might well have a role in the treatment of PA-infected patients with cystic fibrosis. Therefore, this AlgL mutant could be co-administered with antibiotics, possibly using a nebulizer, for this purpose.

## Supporting Information

S1 Fig Tracked geometrical root-mean square deviations (RMSD) and distances in the MD-simulated AlgL-alginate binding structure. (TIF)

S2 Fig Tracked distances in the MD-simulated AlgL-alginate binding structure. (TIF)

S3 Fig Tracked distances in the MD-simulated AlgL-alginate binding structure. (TIF)

S4 Fig As tracked along the MD trajectory, the shortest distances between the positively charged head group at the K319 side chain of AlgL and the carbonyl oxygen atoms (O10 and O11) from the M3 unit of the substrate, and the hydroxyl oxygen atom (O13) at the M2 unit of the substrate. (TIF)

S5 Fig Tracked distances in MD-simulated AlgL-alginate binding structure. (TIF)

## Abbreviations

AFM: atomic force microscope
AlgL: alginate lyases
AvAlgL: *Azotobacter vinelandii* alginate lyase
CRPA: ciprofloxacin-resistant *Pseudomonas aeruginosa*
DTT: dithiothreitol
EDTA: ethylenediamine-*N*,*N*,*N’*,*N’*-tetraacetic acid
MD: molecular dynamics
PA: *Pseudomonas aeruginosa*
PaAlgL: *Pseudomonas aeruginosa* alginate lyase
PBS: phosphate-buffered saline
PMSF: phenylmethanesulfonyl fluoride
PT: piperacillin/tazobactam
RMSD: root-mean square deviation
SDS: sodium dodecylsulfate
SEM: scanning electron microscope
TSB: tryptone soy broth

## References

[1] Wingender J, Strathmann M, Rode A, Leis A, Flemming HC. Isolation and biochemical characterization of extracellular polymeric substances from *Pseudomonas aeruginosa*. Methods Enzymol. 2001; 336: 302–314. 11398408 10.1016/s0076-6879(01)36597-7

[2] Watnick PI, Kolter R. Steps in the development of a *Vibrio cholerae* El Tor biofilm. Mol Microbiol. 1999; 34: 586–595. 10564499 10.1046/j.1365-2958.1999.01624.x PMC2860543

[3] Avila M, Ojcius DM, Yilmaz O. The oral microbiota: living with a permanent guest. DNA Cell Biol. 2009; 28: 405–411. 10.1089/dna.2009.0874 19485767 PMC2768665

[4] Hoiby N, Doring G, Schiotz PO. The role of immune complexes in the pathogenesis of bacterial infections. Annu Rev Microbiol. 1986; 40: 29–53. 2946261 10.1146/annurev.mi.40.100186.000333

[5] Bjarnsholt T, Jensen PO, Fiandaca MJ, Pedersen J, Hansen CR, Andersen CB, et al *Pseudomonas aeruginosa* biofilms in the respiratory tract of cystic fibrosis patients. Pediatr Pulmonol. 2009; 44: 547–558. 10.1002/ppul.21011 19418571

[6] Hall-Stoodley L, Hu FZ, Gieseke A, Nistico L, Nguyen D, Hayes J, et al Direct detection of bacterial biofilms on the middle-ear mucosa of children with chronic otitis media. JAMA. 2006; 296: 202–211. 16835426 10.1001/jama.296.2.202 PMC1885379

[7] Sanderson AR, Leid JG, Hunsaker D. Bacterial biofilms on the sinus mucosa of human subjects with chronic rhinosinusitis. Laryngoscope 2006; 116: 1121–1126. 16826045 10.1097/01.mlg.0000221954.05467.54

[8] Gristina AG, Oga M, Webb LX, Hobgood CD. Adherent bacterial colonization in the pathogenesis of osteomyelitis. Science 1985; 228: 990–993. 4001933 10.1126/science.4001933

[9] Trampuz A, Zimmerli W. Prostetic joint infections: update in diagnosis and treatment. Swiss Med Wkly. 2005; 135: 243–251. 15965826 10.4414/smw.2005.10934

[10] Del Pozo JL, Patel R. Infection associated with prostetic joints. N Engl J Med. 2009; 361: 787–794. 10.1056/NEJMcp0905029 19692690 PMC2850113

[11] Kirketerp-Moller K, Jensen PO, Fazli M, Madsen KG, Pedersen J, Moser C, et al Distribution, and ecology of bacteria in chronic wounds. J Clin Microbiol. 2008; 46: 2717–2722. 10.1128/JCM.00501-08 18508940 PMC2519454

[12] Bjarnsholt T, Kirketerp-Moller K, Jensen PO, Madsen KG, Phipps R, Krogfelt K, Why chronic wounds won’t heal: a novel hypothesis. Wound Repair Regen. 2008; 16: 2–10. 10.1111/j.1524-475X.2007.00283.x 18211573

[13] Taconelli E, Smith G, Hieke K, Lafuma A, Bastide P. Epidemiology, medical outcomes and costs of catheter-related bloodstream infection in intensive care units of four European countries: litterature- and registry-based estimates. J Hosp Infect. 2009; 72: 97–103. 10.1016/j.jhin.2008.12.012 19246122

[14] Jang CH, Cho YB, Choi CH. Structural features of tympanostomy tube biofilm formation in ciprofloxacin-resistant *Pseudomonas otorrhea*. Int J Pediatr Otorhinolaryngol. 2007; 71: 591–595. 17239963 10.1016/j.ijporl.2006.12.005

[15] Yamasaki M, Moriwaki S, Miyake O, Hashimoto W, Murata K, Mikami B. Structure and function of a hypothetical *Pseudomonas aeruginosa* protein PA1167 classified into family PL-7. J Biol Chem. 2004; 279(30): 31863–31872. 15136569 10.1074/jbc.M402466200

[16] Nivens DE, Ohman DE, Williams J, Franklin MJ. Role of alginate and its O acetylation in formation of *Pseudomonas aeruginosa* microcolonies and biofilms. J Bacteriol. 2001; 183:1047–1057. 11208804 10.1128/JB.183.3.1047-1057.2001 PMC94973

[17] Pier GB, Coleman F, Grout M, Franklin M, Ohman DE. Role of alginate O acetylation in resistance of mucoid *Pseudomonas aeruginosa* to opsonic phagocytosis. Infect Immun. 2001; 69: 1895–1901. 11179370 10.1128/IAI.69.3.1895-1901.2001 PMC98099

[18] Hay ID, Gatland K, Campisano A, Jordens JZ, Rehm BHH. Impact of Alginate Overproduction on Attachment and Biofilm Architecture of a Supermucoid *Pseudomonas aeruginosa* Strain. Appl Environ Microbiol. 2009; 75: 6022–6025. 10.1128/AEM.01078-09 19648373 PMC2747843

[19] Bayer AS, Park S, Ramos MC, Nast CC, Eftikhar F, Schiller NL. Effect of alginate on the natural history and antibiotic therapy of experimental endocarditis caused by mucoid *Pseudomonas aeruginosa*. Infect Immun. 1992; 60: 3979–3985. 1398909 10.1128/iai.60.10.3979-3985.1992 PMC257426

[20] Mai GT, McCormack JG, Seow WK, Pier GB, Jackson LA, Thong YH. Inhibition of Adherence of Mucoid Pseudomonas-Aeruginosa by Aalginase, Specipic Monoclonal-Antibodies, and Antibiotics. Infect Immun. 1993; 61: 4338–4343. 8406822 10.1128/iai.61.10.4338-4343.1993 PMC281163

[21] Alkawash MA, Soothill JS, Schiller NL. Alginate lyase enhances antibiotic killing of mucoid *Pseudomonas aeruginosa* in biofilms. APMIS. 2006; 114: 131–138. 16519750 10.1111/j.1600-0463.2006.apm_356.x

[22] Grasdalen H. High-field, 1H-n.m.r. spectroscopy of alginate: sequential structure and linkage conformations. Carbohydr Res. 1983; 118: 255–260.

[23] Grasdalen H, Larsen B, Smidsrod O. 13C-N.M.R. studies of monomeric composition and sequence in alginate. Carbohydr Res. 1981; 89: 179–191.

[24] Davidson JW, Lawson CJ, Sutherland IW. Localization of O-acetyl groups in bacterial alginate. J Gen Microbiol. 1977; 98: 603–606. 10.1099/00221287-98-1-223 13144

[25] Franklin MJ, Ohman DE. Identification of *algF* in the alginate biosynthetic gene cluster of *Pseudomonas aeruginosa* which is required for alginate acetylation. J Bacteriol. 1993; 175: 5057–5065. 8394313 10.1128/jb.175.16.5057-5065.1993 PMC204972

[26] Skjak-Braek G, Grasdalen H, Larsen B. Monomer sequence and acetylation pattern in some bacterial alginates. Carbohydr Res. 1986; 154: 239–250. 3098421 10.1016/s0008-6215(00)90036-3

[27] Leid JG, Willson CJ, Shirtliff ME, Hassett DJ, Parsek MR, Jeffers AK. The exopolysaccharide alginate protect *Pseudomonas aeruginosa* biofilm bacteria from IFNγ-mediated macrophage killing. J Immunol. 2005; 175: 7512–7518. 16301659 10.4049/jimmunol.175.11.7512

[28] Kotra LP, Amro NA, Leu GY, Mobashery S. Visualizing bacteria at high resolution. ASM News 2000; 66: 675–681.

[29] Case DA, Darden TA, Cheatham TE, Simmerling CL III, Wang J, Duke RE, et al AMBER 12, University of California, San Francisco 2012.

[30] Huang X, Zheng F, Zhan C-G. Binding structures and energies of the human neonatal Fc receptor with human Fc and its mutants by molecular modeling and dynamics simulations. Mol Biosyst. 2013; 9: 3047–3058. 10.1039/c3mb70231f 24057047 PMC3834255

[31] Huang X, Zheng G, Zhan C-G. Microscopic binding of M5 muscarinic acetylcholine receptor with antagonists by homology modeling, molecular docking, and molecular dynamics simulations. J Phys Chem B. 2012; 116: 532–541. 10.1021/jp210579b 22185605 PMC3257414

[32] Mikami B, Ban M, Suzuki S, Yoon HJ, Miyake O, Yamasaki M, et al Induced-fit motion of a lid loop involved in catalysis in alginate lyase A1-III. Acta Crystallogr D Biol Crystallogr. 2012; 68: 1207–1216. 10.1107/S090744491202495X 22948922

[33] Henikoff S, Henikoff JG. Amino-acid substitution matrices from protein blocks. Proc Natl Acad Sci USA. 1992; 89: 10915–10919. 1438297 10.1073/pnas.89.22.10915 PMC50453

[34] Thompson JD, Higgins DG, Gibson TJ. CLUSTAL W: improving the sensitivity of progressive multiple sequence alignment through sequence weighting, position-specific gap penalties, and weight matrix choice. Nucleic Acids Res. 1994; 22: 4673–4680. 7984417 10.1093/nar/22.22.4673 PMC308517

[35] Frisch MJ, Trucks GW, Schlegel HB, Scuseria GE, Robb MA, Cheeseman JR, et al Gaussian 03, revision A.1; Gaussian, Inc: Pittsburgh, PA 2003.

[36] Duan Y, Wu C, Chowdhury S, Lee MC, Xiong G, Zhang W, et al A point-charge force field for molecular mechanics simulations of proteins based on condensed-phase quantum mechanical calculations. J Comput Chem. 2003; 24: 1999–2012. 14531054 10.1002/jcc.10349

[37] Wang J, Wolf RM, Caldwell JW, Kollman PA, Case DA. Development and testing of a general amber force field. J Comput Chem. 2004; 25: 1157–1174. 15116359 10.1002/jcc.20035

[38] Berendsen HJC, Postma JPM, van Gunsteren WF, DiNola A, Haak JR. Molecular dynamics with coupling to an external bath. J Chem Phys. 1984; 81: 3684–3690.

[39] Darden T, York D, Pedersen L. Particle mesh Ewald: an *N*∙log(*N*) method for Ewald sums in large systems. J Chem Phys. 1993; 98: 10089–10092.

[40] Ryckaert JP, Ciccotti G, Berendsen HJC. Numerical integration of the Cartesian equations of motion of a system with constraints: Molecular dynamics of n-alkanes. J Comput Phys. 1997; 23: 327–341.

[41] Veeranagouda Y, Basavaraja C, Bae H-S, Liu K-H, Lee K. Augmented production of poly-β-D-mannuronate and its acetylated forms by *Pseudomonas*. Process Biochem. 2011; 46: 328–334.

[42] Braman J, Papworth C, Greener A. Site-directed mutagenesis using double-stranded plasmid DNA templates. Methods in Molecular Biology 1996; 57: 31–44. 8849992 10.1385/0-89603-332-5:31

[43] Weissbach A, Hurwitz J. The formation of 2-keto-3-deoxyheptonic acid in extracts of *Escherichia coli* B. J Biol Chem. 1959; 234: 705–709. 13654246

[44] Garcia-Saenz MC, Arias-Puente A, Fresnadillo-Martinez MJ, Matilla-Rodriguez A. *In vitro* adhesion of *Staphylococcus epidermidis* to intraocular lenses. J Cataract Refract Surg. 2000; 26: 1673–1679. 11084278 10.1016/s0886-3350(00)00483-1

[45] Rehm BH, Ertesvag H, Valla S. A new *Azotobacter vinelandii* mannuronan C-5-epimerase gene is part of an *alg* gene cluster physically organized in a manner similar to that in *Pseudomonas aeruginosa*. J Bacteriol. 1996; 178(20): 5884–5889. 8830682 10.1128/jb.178.20.5884-5889.1996 PMC178442

[46] MacDonald LC, Berger BW. Insight into the role of substrate-binding residues in conferring substrate specificity for the multifunctional polysaccharide lyase Smlt1473. J Biol Chem. 2014; 289: 18022–18032. 10.1074/jbc.M114.571299 24808176 PMC4140301

[47] Blackledge MS, Worthington RJ, Melander C. Biologically inspired strategies for combating bacterial biofilms. Curr Opin Pharmacol. 2013; 13: 699–706. 10.1016/j.coph.2013.07.004 23871261 PMC3795836

[48] Yang L, Liu Y, Wu H, Song Z, Hoiby N, Molin S, et al Combating biofilms. FEMS Immunol Med Microbiol. 2012; 65: 146–157. 10.1111/j.1574-695X.2011.00858.x 22066868

[49] Masak J, Cejkova A, Schreiberova O, Rezanka T. *Pseudomonas* biofilms: possibilities of their control. FEMS Microbiol Ecol. 2014; 89: 1–14. 10.1111/1574-6941.12344 24754832

[50] Frederiksen B, Pressler T, Hansen A, Koch C, Hoiby N. Effect of aerosolized rhDNase (Pulmozyme) on pulmonary colonization in patients with cystic fibrosis. Acta Paediatr. 2006; 95: 1070–1074. 16938752 10.1080/08035250600752466

[51] Chaignon P, Sadovskaya I, Ragunah Ch, Ramasubbu N, Kaplan JB, Jabbouri S. Susceptibility of staphylococcal biofilms to enzymatic treatments depends on their chemical composition. Appl Microbiol Biotechnol. 2007; 75: 125–132. 17221196 10.1007/s00253-006-0790-y

