# Supplementary figures and images for: Modeling and Re-Engineering of Azotobacter vinelandii Alginate Lyase to Enhance Its Catalytic Efficiency for Accelerating Biofilm Degradation
Source: PLoS One. 2016 Jun 2;11(6):e0156197. doi: 10.1371/journal.pone.0156197 (PMC4890793; doi:10.1371/journal.pone.0156197)

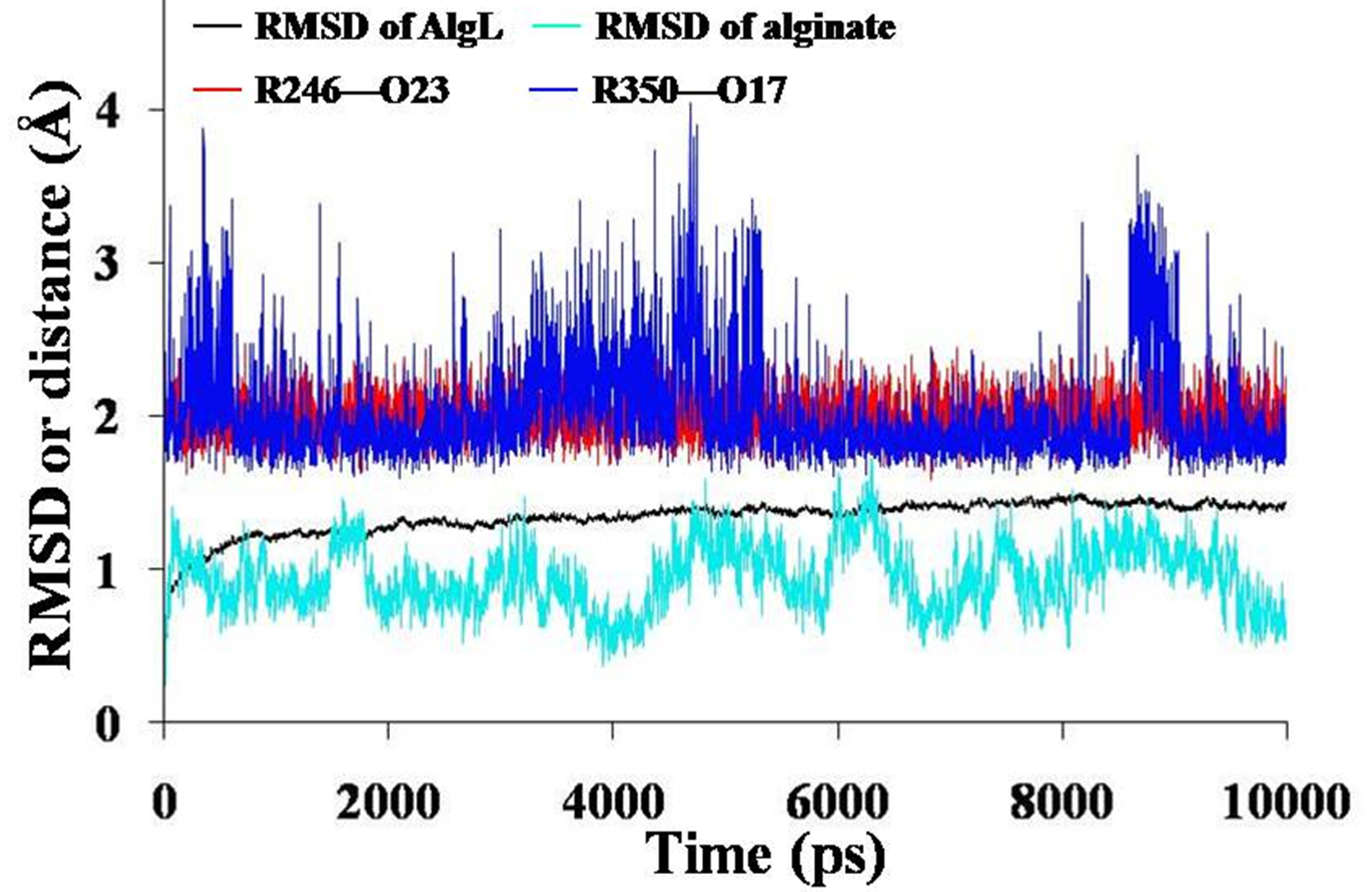

Supplement: S1 Fig — (TIF) [file pone.0156197.s001.TIF]

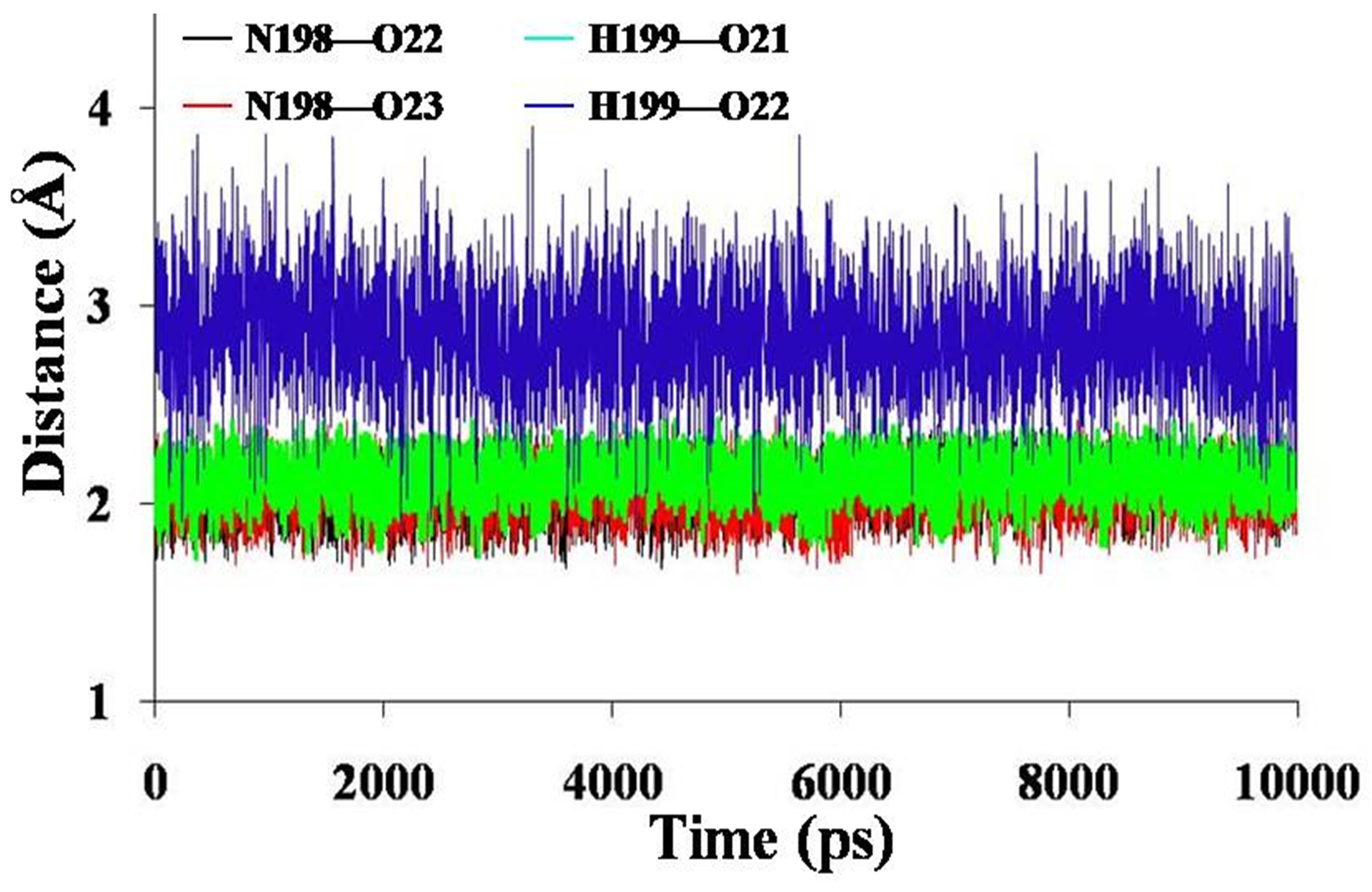

Supplement: S2 Fig — (TIF) [file pone.0156197.s002.TIF]

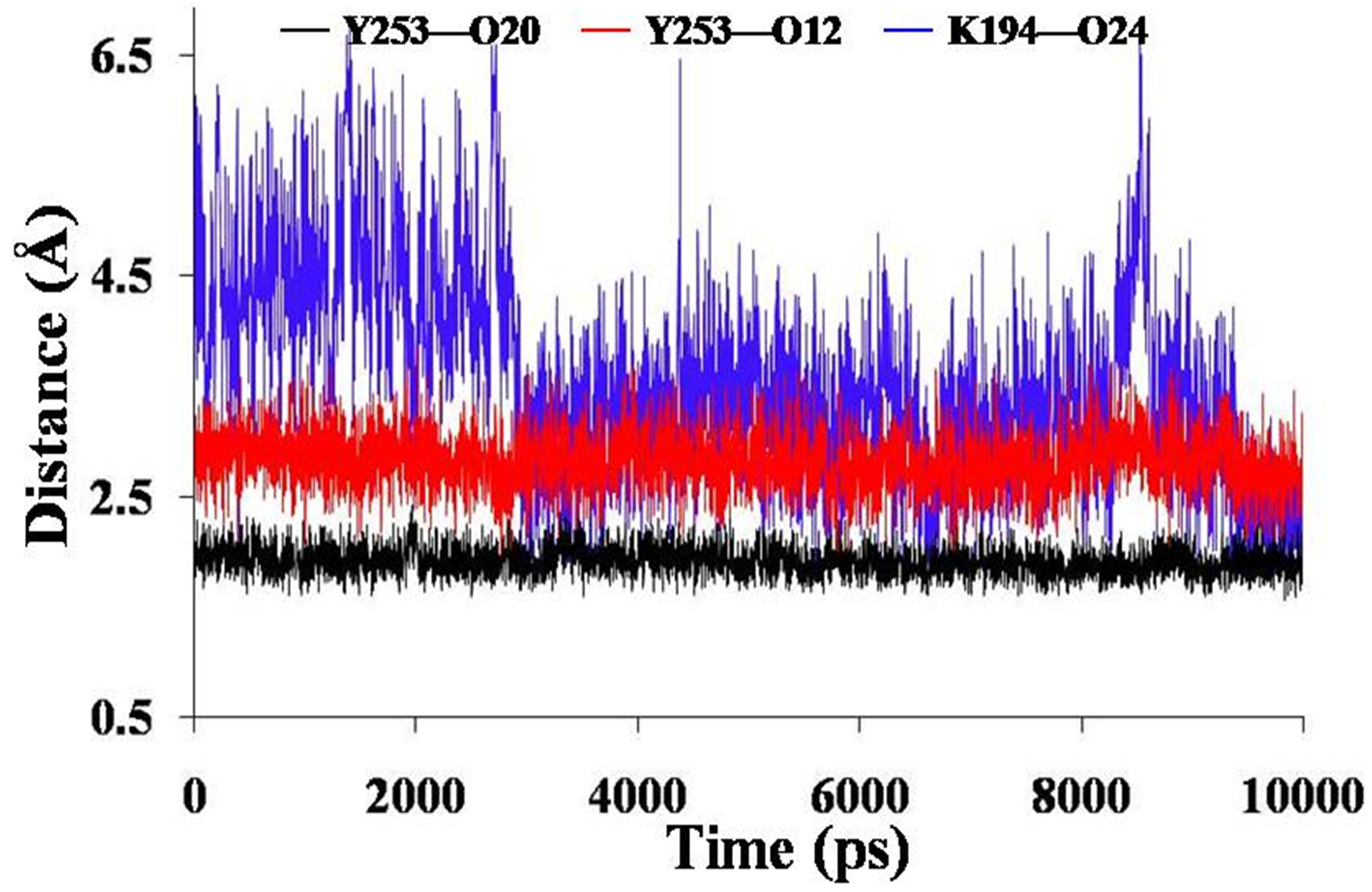

Supplement: S3 Fig — (TIF) [file pone.0156197.s003.TIF]

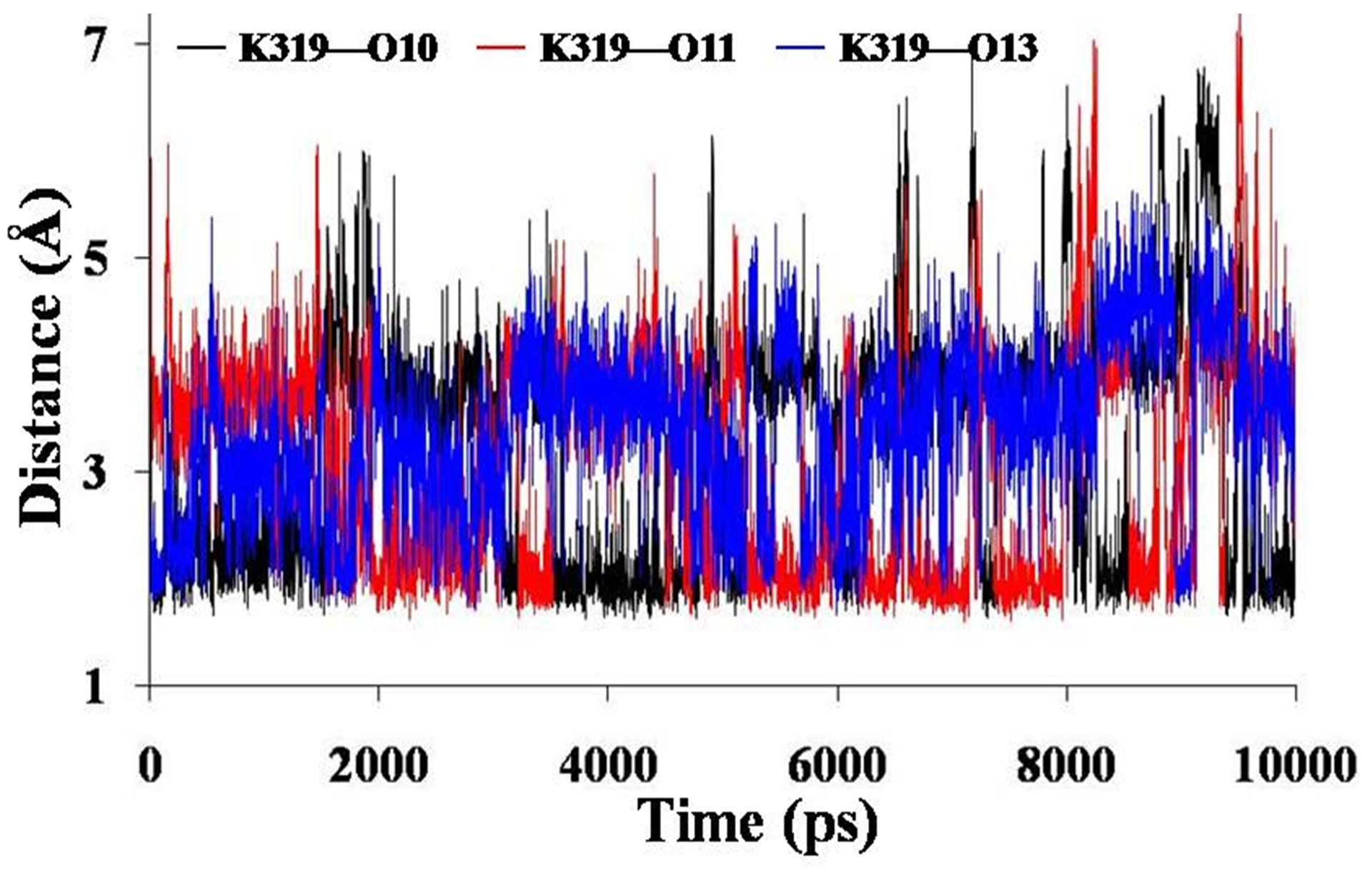

Supplement: S4 Fig — (TIF) [file pone.0156197.s004.TIF]

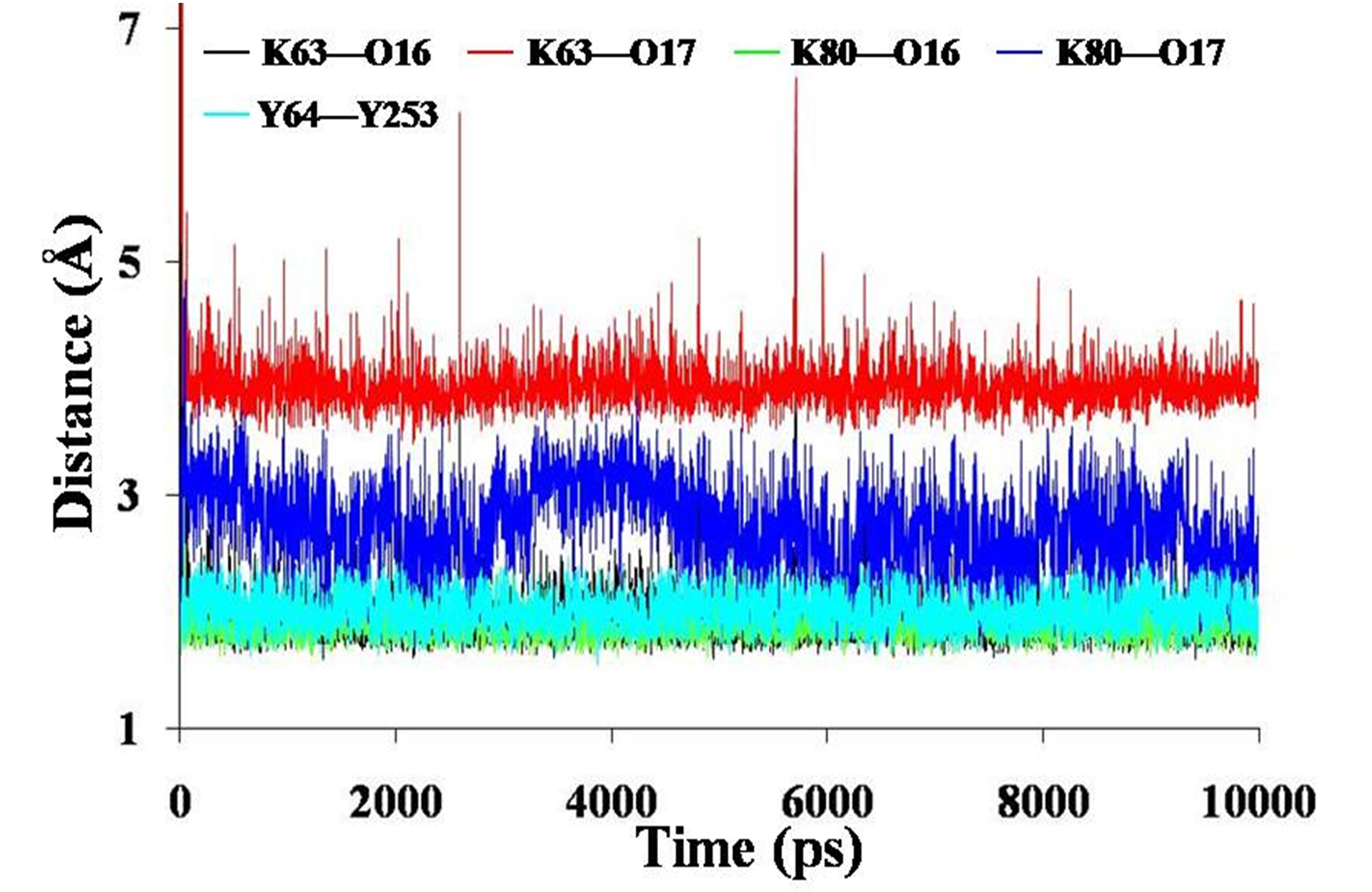

Supplement: S5 Fig — (TIF) [file pone.0156197.s005.TIF]
